# A Critical Assessment of the Microorganisms Proposed to be Important to Enhanced Biological Phosphorus Removal in Full-Scale Wastewater Treatment Systems

**DOI:** 10.3389/fmicb.2017.00718

**Published:** 2017-04-27

**Authors:** Mikkel Stokholm-Bjerregaard, Simon J. McIlroy, Marta Nierychlo, Søren M. Karst, Mads Albertsen, Per H. Nielsen

**Affiliations:** Center for Microbial Communities, Department of Chemistry and Bioscience, Aalborg UniversityAalborg, Denmark

**Keywords:** 16S rRNA amplicon sequencing, Accumulibacter, activated sludge, EBPR, GAO, PAO, *Micropruina*, *Tetrasphaera*

## Abstract

Understanding the microbiology of phosphorus (P) removal is considered essential to knowledge-based optimization of enhanced biological P removal (EBPR) systems. Biological P removal is achieved in these systems by promoting the growth of organisms collectively known as the polyphosphate accumulating organisms (PAOs). Also considered important to EBPR are the glycogen accumulating organisms (GAOs), which are theorized to compete with the PAOs for resources at the expense of P removal efficiency. Numerous studies have sought to identify the PAOs and their GAOs competitors, with several candidates proposed for each over the last few decades. The current study collectively assessed the abundance and diversity of all proposed PAOs and GAOs in 18 Danish full-scale wastewater treatment plants with well-working biological nutrient removal over a period of 9 years using 16S rRNA gene amplicon sequencing. The microbial community structure in all plants was relatively stable over time. Evidence for the role of the proposed PAOs and GAOs in EBPR varies and is critically assessed, in light of their calculated amplicon abundances, to indicate which of these are important in full-scale systems. Bacteria from the genus *Tetrasphaera* were the most abundant of the PAOs. The “*Candidatus* Accumulibacter” PAOs were in much lower abundance and appear to be biased by the amplicon-based method applied. The genera *Dechloromonas, Microlunatus*, and *Tessaracoccus* were identified as abundant putative PAO that require further research attention. Interestingly, the actinobacterial *Micropruina* and sbr-gs28 phylotypes were among the most abundant of the putative GAOs. Members of the genera *Defluviicoccus, Propionivibrio*, the family Competibacteraceae, and the spb280 group were also relatively abundant in some plants. Despite observed high abundances of GAOs (periodically exceeding 20% of the amplicon reads), P removal performance was maintained, indicating that these organisms were not outcompeting the PAOs in these EBPR systems. Phylogenetic diversity within each of the PAOs and GAOs genera was observed, which is consistent with reported metabolic diversity for these. Whether or not key traits can be assigned to sub-genus level clades requires further investigation.

## Introduction

Wastewater treatment using the enhanced biological phosphorus removal (EBPR) process is capable of achieving low effluent phosphorus (P) concentrations without addition of chemical precipitants. EBPR systems achieve this by utilizing the ability of polyphosphate accumulating organisms (PAOs) to take up and store excessive amounts of P. Hence, compared to chemical precipitation, EBPR is a sustainable, effective, and economical process (Seviour et al., 2003). In addition, P is an essential nutrient used in fertilizers and is a limited non-renewable resource (Gilbert, 2009). Thus, P-rich sludge from EBPR plants is seen as a high quality, sustainable, and economical alternative source of P (Jeanmaire and Evans, 2001; Molinos-Senante et al., 2011). As EBPR systems are at times subject to failure, or periods of low efficiency, an understanding of the microbiology that underpins the process is important for knowledge-based optimization. As such, many studies on the identity, diversity, and physiology of PAOs and their proposed competitors, the glycogen accumulating organisms (GAOs), have been carried out (Oehmen et al., 2007).

Early PAO metabolic models were proposed to explain the key transformations of the dynamic EBPR environment, where biomass is cycled between carbon rich anaerobic (feast) and carbon deficient aerobic (famine) zones (Comeau et al., 1986; Wentzel et al., 1986; Mino et al., 1987). Volatile fatty acids (VFAs) are stored as polyhydroxyalkanoates (PHAs) under anaerobic conditions, where aerobically stored polyphosphate and glycogen provide the energy and reducing equivalents required. In the subsequent aerobic phase, stored PHAs are utilized for growth and regeneration of glycogen and polyphosphate stores (Oehmen et al., 2007). Substantial effort has been made to identify organisms that conform to these proposed models. Early culture-based approaches identified several activated sludge isolates, which accumulated excess polyphosphate. Of these isolates, members of the genus *Acinetobacter* received considerable research attention but were never shown to conform to PAO models (Jenkins and Tandoi, 1991; Tandoi et al., 1998a; Seviour et al., 2003), and their low abundance in full-scale systems (Wagner et al., 1994) appears to be maintained by immigration with the influent rather than active growth (Saunders et al., 2016). The betaproteobacterial isolate *Lampropedia hyalina* was shown to behave according to the proposed PAO models, with anaerobic storage of acetate as PHA, although associated P cycling was much lower than for EBPR biomass (Stante et al., 1997) and its abundance in full-scale EBPR systems is not known. The role of other isolates has not been assessed further than their ability to store polyphosphate. These include *Tessarococcus bendigoensis* (Maszenan et al., 1999b), *Friedmaniella* spp. (Maszenan et al., 1999a), *Quatrionicoccus australiensis* (Maszenan et al., 2002), *Gemmatimonas aurantiaca* (Zhang et al., 2003), and *Malikia granosa* (Spring et al., 2005).

The advent of molecular methods revolutionized our understanding of EBPR microbiology generally. In particular, fluorescence *in situ* hybridization (FISH) is applied to assess *in situ* abundances and, when coupled with other methods, key metabolic traits can be demonstrated (Wagner and Haider, 2012). Typically, FISH has been applied with microautoradiography (MAR; Nierychlo et al., 2015) and fluorescent staining methods (Serafim et al., 2002) to demonstrate *in situ* uptake of carbon and P and, PHA and polyphosphate storage, respectively. Raman-spectroscopy and novel staining methods should also allow *in situ* analyses of glycogen (Huang et al., 2007; Majed and Gu, 2010; Mesquita et al., 2013). Such methods have been applied to at least partially demonstrate the PAO phenotype *in situ* for “*Candidatus* Accumulibacter spp.” (Hesselmann et al., 1999; Crocetti et al., 2000; Kong et al., 2004), *Tetrasphaera* spp. (Kong et al., 2005; Nguyen et al., 2011), *Microlunatus* spp. (Kawaharasaki et al., 1999; Beer et al., 2006), *Dechloromonas* spp. (Kong et al., 2007; Günther et al., 2009), *Pseudomonas* spp. (Günther et al., 2009), and “*Candidatus* Accumulimonas spp.” (formerly “*Candidatus* Halomonas phosphatis”; Nguyen et al., 2012).

Changing the way we define PAOs in EBPR, the characterization of the actinobacterial genus *Tetrasphaera* found that these organisms cycle polyphosphate, but without PHA accumulation, which is not consistent with the original PAO models. Instead, they seem to mainly utilize sugars and amino acids through a fermentative metabolism, where polyphosphate supplements anaerobic energy requirements (Kong et al., 2005; Nguyen et al., 2011; Kristiansen et al., 2013). Members of the related actinobacterial genus *Microlunatus* also appear to possess a similar physiology (Nakamura et al., 1995a,b; Santos et al., 1999; Kawakoshi et al., 2012). Therefore, in the context of EBPR, the PAO is now more broadly defined to cover any organism utilizing aerobically stored polyphosphate to energize anaerobic carbon uptake (Seviour and McIlroy, 2008).

The GAO phenotype model is similar to that of the traditional “*Ca.* Accumulibacter” PAO phenotype, except polyphosphate is not cycled, which is compensated by a reliance on glycogen as energy source under anaerobic conditions (Liu et al., 1994; Mino et al., 1995). As they compete with the PAO for resources without contributing to P removal, their proliferation is widely considered to be at the expense of EBPR efficiency (Seviour et al., 2003; Oehmen et al., 2007). As with the PAO, early culture-based studies sought to identify organisms conforming to the proposed GAO model. Because of the frequent association of EBPR failure and the proliferation of tetrad-forming organisms (TFOs), there was a focus on organisms with this morphology accumulating PHAs and glycogen but not polyphosphate—originally referred to as the “G-bacteria,” named for their suggested affinity for glucose assimilation and defined by their TFO morphology (Cech and Hartman, 1993; Seviour et al., 2000). Several isolates from poorly performing systems were suggested. Of these isolates, the GAO phenotype has only been confirmed for *Defluviicoccus vanus* (Wong and Liu, 2007). *Amaricoccus kaplicensis*, the original G-bacteria, has not been shown to accumulate substrates under anaerobic conditions, which is key to the GAO phenotype (Falvo et al., 2001). Little is known of the potential importance of other isolates, including the actinobacterial *Nakamurella multipartita* (Yoshimi et al., 1996), *Micropruina glycogenica* (Shintani et al., 2000), and *Kineosphaera limosa* (Liu et al., 2000, 2002).

The application of molecular methods has also allowed the study of the putative GAO *in situ* where the phenotype has been at least partially confirmed for: the actinobacterial genus *Micropruina* and closely related sbr-gs28 taxon (Kong et al., 2001), the gammaproteobacterial family Competibacteraceae (Kong et al., 2006) and CCM19a phylotype (probe Gam445) (Kong et al., 2007), the alphaproteobacterial genus *Defluviicoccus* (Wong et al., 2004; Meyer et al., 2006; Burow et al., 2007; Wong and Liu, 2007; McIlroy et al., 2010), and the betaproteobacterial genera *Propionivibrio* (Albertsen et al., 2016), and Spb280 (probe Bet65; Kong et al., 2007).

The PAO and GAO phenotypes appear to encompass both phylogenetically and metabolically diverse organisms. Importantly, most studies into the PAO and GAO are based on lab-scale studies with VFAs, such as acetate, as the sole carbon source. In these systems, the “*Ca.* Accumulibacter” PAO, and the Competibacteraceae and *Defluviicoccus* GAO, are almost exclusively found, and are therefore widely considered to be the most important groups to EBPR. However, it is still unclear which of the putative PAOs and GAOs are actually the key organisms in full-scale plants, noting that these systems will harbor a much higher phylogenetic and phenotypic diversity relative to the lab-scale environment. For example, large-scale FISH surveys have revealed that *Tetrasphaera* spp. are the most abundant genus in Danish full-scale EBPR systems (Mielczarek et al., 2013), which brings into question the belief that the “*Ca.* Accumulibacter” are the most important PAO phylotype. Developments in protocols and technology for the amplicon sequencing of the 16S rRNA gene now allow high-throughput analyses of the microbial community composition in full-scale systems (Zhang et al., 2012; Saunders et al., 2016). The on-going Microbial Database of Activated Sludge (MiDAS) initiative implements this technology to survey the bacterial communities of 18 Danish wastewater treatment plants (WWTPs) over a period of 9 years^1^ (McIlroy et al., 2015b).

The current study will, for the first time, collectively assess the relative abundance and distribution of all suggested PAO and GAO in Danish full-scale EBPR WWTPs, using the extensive MiDAS survey data. In this article, the available literature for each suggested PAO and GAO is critically assessed, along with their relative amplicon-based abundance, to indicate their potential importance in full-scale systems. In doing so, the study identifies key organisms and questions for the focus of future studies into the microbiology of EBPR.

## Materials and Methods

### Treatment Plants and Sampling

Sampling and investigation of the microbial populations in the activated sludge biomass from 18 plants with EBPR was done within the MiDAS project (McIlroy et al., 2015b). The plants were sampled up to four times a year from 2006 to 2014 giving a total of 414 samples. They were sampled February, May, August, and October. All samples were taken from the aeration tank and sent by over night mail to our laboratory for processing.

The WWTPs investigated included the following variations in design and operation: +/- primary settling, +/- digester, alternating/recirculation flow, +/- return sludge sidestream hydrolysis (RSS), different industrial loading, and different type and amount of external carbon source added (**Table 1**). All plants were municipal WWTPs located in Denmark treating primarily household wastewater with contributions from industry of 5–75% of the influent chemical oxygen demand (COD). All plants had EBPR and supplementary chemical P removal. Median temperature was similar in all plants and ranged from 9°C in the winter and 18°C in the summer. pH was stable at 7.2 (±0.3). The median suspended solids (SS) was 4.9 g L^-1^ with some seasonal variation from 4.4 g L^-1^ in summer to 5.1 g L^-1^ in winter. Median influent total COD, total nitrogen (N), and total P after primary treatment (if present) were 528, 42, and 7.3 mg L^-1^.

**Table 1:** Design and operation of wastewater treatment plants (WWTPs) included in the survey.

WWTP	Configuration	Industrial waste (% COD)	Primary settling	Digester	Return sludge side stream hydrolysis	P removal mean (%)
Bjergmarken	Alternating	20	No	Yes	No	94 ± 3
Boeslum	Recirculation	5	No	No	No	98 ± 1
Egaa	Recirculation	40	No	No	Yes	97 ± 1
Ejby	Alternating	49–55	Yes	Yes	No	95 ± 8
Fredericia	Recirculation	75	No	Yes	No	90 ± 8
Haderslev	Alternating	5	No	No	Yes	91 ± 6
Hirtshals	Alternating	60–70	No	No	No	98 ± 1
Hjørring	Recirculation	30	Yes	Yes	No	94 ± 7
Lundtofte	Alternating	5	Yes	Yes	No	83 ± 10
Odense NE	Alternating	20	Yes	Yes	No	97 ± 1
Randers	Recirculation	5	Yes	Yes	Yes	91 ± 4
Ribe	Recirculation	20	No	No	Yes	95 ± 3
Ringkøbing	Alternating	10	Yes	Yes	Yes	96 ± 2
Skive	Recirculation	20–65	No	No	Yes	94 ± 4
Søholt	Alternating	35	Yes	Yes	Yes	98 ± 1
Åby	Alternating	20	No	Yes	No	96 ± 3
Aalborg E	Alternating	10	No	Yes	Yes	92 ± 6
Aalborg W	Alternating	30	Yes	Yes	Yes	92 ± 4

*COD, chemical oxygen demand.*

All plants were operated under the same minimum effluent quality regulations of 75 mg L^-1^ total COD, 8 mg L^-1^ total N, and 1.5 mg L^-1^ total P. The actual effluent quality was, however, better, with median effluent COD, N, and P of 25, 4, and 0.3 mg L^-1^, respectively. P removal efficiency was calculated as the percentage of the removed total P by comparing monthly mean values of influent and effluent concentrations.

### DNA Extraction and Amplicon Sequencing

DNA extraction was conducted using the FastDNA spin kit for soil (MP Biomedicals) according to the manufactures instructions, except the bead beating was increased to 4 × 40 s at 6 m/s using a FastPrep FP120 (MP Biomedicals). The procedure for bacterial 16S rRNA amplicon sequencing targeting the V1-3 variable region was modified from Caporaso et al. (2010). Briefly, 10 ng of extracted DNA was used as template and the polymerase chain reaction (PCR) (25 μL) contained dNTPs (400 nM of each), MgSO_4_ (1.5 mM), Platinum^®^ Taq DNA polymerase high fidelity (HF) (2 mU), 1× Platinum^®^ High Fidelity buffer (Thermo Fisher Scientific) and a pair of barcoded library adaptors (400 nM). V1-3 primers: 27F AGAGTTTGATCCTGGCTCAG and 534R ATTACCGCGGCTGCTGG (Chen et al., 2010). Thermo cycler settings: initial denaturation at 95°C for 2 min, 30 cycles of 95°C for 20 s, 56°C for 30 s, 72°C for 60 s, and final elongation at 72°C for 5 min. All PCR were run in duplicate and pooled afterward. The amplicon libraries were purified using the Agencourt^®^ AMpure XP bead protocol (Beckmann Coulter, Brea, CA, USA) with the following exceptions: the sample/bead solution ratio was 5/4 and the purified DNA was eluted in 33 μL nuclease-free water. Library concentration was measured with Quant-iT^TM^ HS DNA Assay (Thermo Fisher Scientific) and quality validated with a Tapestation 2200 using D1K ScreenTapes (Agilent). Based on library concentrations and calculated amplicon sizes the samples were pooled in equimolar concentrations and diluted to 4 nM. The library pool was sequenced on a MiSeq (Illumina) using a MiSeq Reagent kit v3 [2 × 300 paired end (PE)] following the procedure in Caporaso et al. (2012), with exception of 10% PhiX control library (Illumina) spike-in and final library loading concentration of 20 pM. A complete detailed DNA extraction and amplicon sequencing protocol can be obtained from^2^ and the optimization of the protocols applied in this study is detailed in Albertsen et al. (2015).

### 16S rRNA Data Processing, Analysis, and Visualization

All sequenced sample libraries were subsampled to 50,000 raw reads and low quality reads removed using Trimmomatic v. 0.32 with the settings SLIDINGWINDOW:1:3 and MINLEN:275 (Lohse et al., 2012). Forward and reverse reads were merged using FLASH v. 1.2.7 (Magoč and Salzberg, 2011), with the settings -m 25 -M 200 and afterward merged reads smaller than 425 bp or larger than 525 bp were discarded. All merged reads were screened for PhiX contamination using usearch v. 7.0.1090 (Edgar, 2010), with standard settings and all matching reads removed. The potential PhiX contamination is due to the use of an un-indexed PhiX as a quality control, which can result in index-carryover from nearby clusters with indexes. The merged reads were de-replicated and formatted for use in the UPARSE workflow (Edgar, 2013). The merged reads were clustered into operational taxonomic units (OTUs) (97% similarity) using the usearch v. 7.0.1090-cluster_otus with default settings. OTU abundance was estimated using the usearch v. 7.0.1090–usearch_global with –id 0.97.

Taxonomy was assigned using the Ribosomal Database Project (RDP) classifier (Wang et al., 2007) as implemented in the parallel_assign_taxonomy_rdp.py script in Quantitative Insights into Microbial Ecology (QIIME) (Caporaso et al., 2010) using MiDAS taxonomy version 2.1. All data analysis and visualizations were conducted using R (R Core Team, 2014) through the Rstudio IDE^3^. OTU counts and associated taxonomic assignments were imported and merged to a phyloseq object (McMurdie and Holmes, 2013) and analyzed using the ampvis R package^4^. The median abundance of the OTUs was determined from the 18 WWTPs with most samples in the database (*n* = 414). The raw V1-3 16S rRNA amplicon sequences are part of the MiDAS dataset^5^ available in European Nucleotide Archive (ENA) with the project ID PRJEB19518. All processed data is included in the ampvis R package as a data object: data (MiDAS_1.20).

It is worth noting that these 16S rRNA gene counts are abundance estimations, and not directly comparable to the true biomass fraction due to DNA extraction biases, primer biases, variation in cell size, and 16S rRNA gene copy number between species (Albertsen et al., 2015).

**FIGURE 1:**
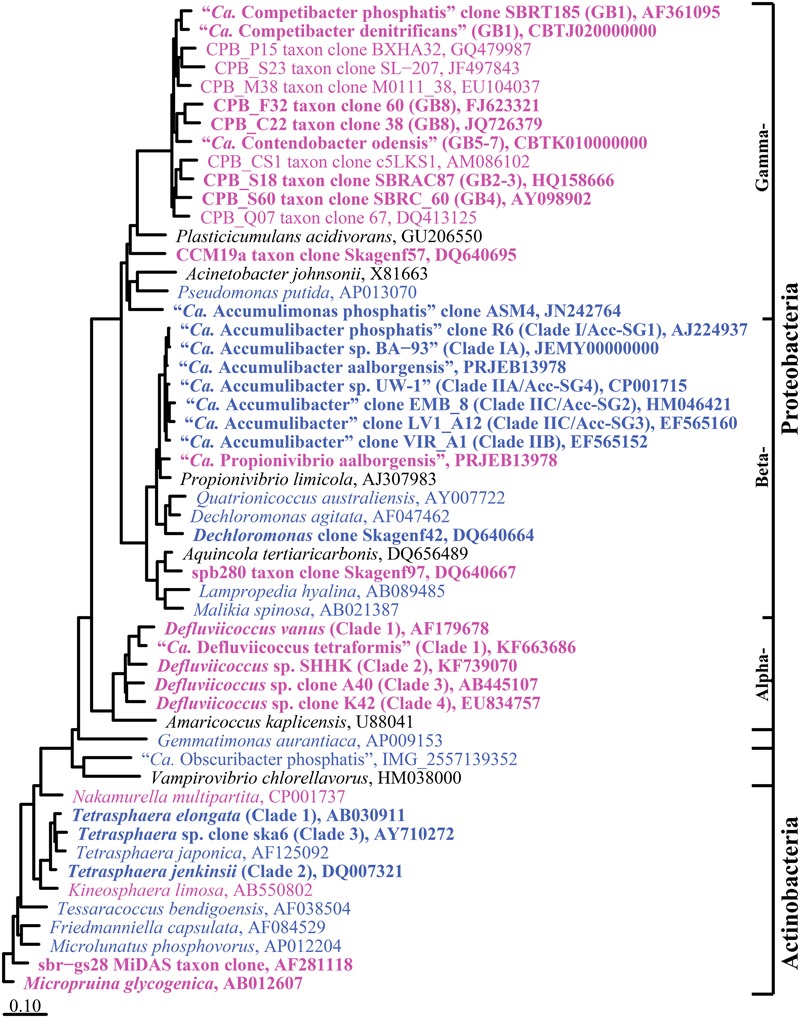
**Maximum-likelihood (PhyML) 16S rRNA gene phylogenetic tree for suggested PAO and GAO in EBPR activated sludge wastewater treatment plants (WWTPs).** Putative PAO sequences are in blue and putative GAO in magenta. Bold typeface indicates the PAO or GAO physiology is supported by *in situ* evidence. Sub-group and clade classifications for the “*Ca.* Accumulibacter spp.” (He et al., 2007; Kim et al., 2010, 2013), Competibacteraceae (Kong et al., 2002; Kim et al., 2011; McIlroy et al., 2015a), and *Defluviicoccus* spp. (Wong et al., 2004; Meyer et al., 2006; McIlroy and Seviour, 2009; Nittami et al., 2009) are taken from previous studies and are given in parenthesis. Brackets to the right indicate the phylogenetic classification of sequences. The tree was prepared using the ARB software (Ludwig et al., 2004) from the MiDAS database (version 2.1) (McIlroy et al., 2015b). Sequences were aligned in the ARB software, trimmed and variable regions excluded with a custom filter (filter by base frequency, 20–100%) leaving 1137 aligned positions. Sequences <1200 bp were added after calculation of the tree with the ARB insert sequences function (AY710272; KF663686). The scale bar represents substitutions per nucleotide base.

The genera proposed to behave as PAO and GAO in EBPR systems are listed in **Tables 2, 3** and their phylogeny is shown in **Figure 1**. Classification is based on the MiDAS taxonomy release 2.1 (McIlroy et al., 2015b), which is a manually curated version of the SILVA 123 NR99 database (Quast et al., 2013), updated to include abundant organisms in wastewater and activated sludge treatment systems not covered by commonly applied public databases. Putative PAO and GAO not covered by other databases include the “*Ca.* Accumulimonas” PAO and the Competibacteraceae, sbr-gs28, spb280, and CCM19a GAO phylotypes.

**Table 2:** Summary of the putative PAO.

Phylum/class	Family	Genus	Reference
Betaproteobacteria	Rhodocyclaceae	“*Ca.* Accumulibacter”	Hesselmann et al., 1999; Crocetti et al., 2000
		*Dechloromonas*	Kong et al., 2007
		*Quatrionicoccus*	Maszenan et al., 2002
	Comamonadaceae	*Lampropedia*	Stante et al., 1997
		*Malikia*	Spring et al., 2005

Gammaproteobacteria	Halomonadaceae	“*Ca.* Accumulimonas”^∗^	Nguyen et al., 2012
	Pseudomonadaceae	*Pseudomonas*	Günther et al., 2009

Actinobacteria	Intrasporangiaceae	*Tetrasphaera*	Kong et al., 2005; Nguyen et al., 2011
	Propionibacteriaceae	*Microlunatus*	Nakamura et al., 1995a
		*Tessaracoccus*	Maszenan et al., 1999b
		*Friedmaniella*	Maszenan et al., 1999a

Gemmatimonadetes	Gemmatimonaceae	*Gemmatimonas*	Zhang et al., 2003

Cyanobacteria	Obscuribacteraceae	“*Ca.* Obscuribacter”	Soo et al., 2014

*^∗^Based on the MiDAS taxonomy (McIlroy et al., 2015b).*

**Table 3:** Summary of the putative GAO.

Phylum/class	Family	Genus	Reference
Alphaproteobacteria	Rhodospirillaceae	*Defluviicoccus* cluster 1^∗^	Wong et al., 2004; Maszenan et al., 2005
		*Defluviicoccus* cluster 2^∗^	Meyer et al., 2006
		*Defluviicoccus* cluster 3^∗^	Nittami et al., 2009
		*Defluviicoccus* cluster 4^∗^	McIlroy and Seviour, 2009

Betaproteobacteria	Rhodocyclaceae	*Propionivibrio*	Albertsen et al., 2016
	Comamonadaceae	spb280^∗^	Kong et al., 2007

Gammaproteobacteria	Competibacteraceae	“*Ca.* Competibacter”^∗^	Crocetti et al., 2002; McIlroy et al., 2015a
		“*Ca.* Contendobacter”^∗^	McIlroy et al., 2014
		CPB_S18^∗^	McIlroy et al., 2015a
		CPB_CS1^∗^	McIlroy et al., 2015a
		CPB_P15^∗^	McIlroy et al., 2015a
		CPB_M38^∗∗^	McIlroy et al., 2015a
		CPB_S23^∗^	McIlroy et al., 2015a
		CPB_Q07^∗^	McIlroy et al., 2015a
		CPB_S60^∗^	McIlroy et al., 2015a
		CPB_C22&F32^∗^	McIlroy et al., 2015a
	CCM19a	CCM19a^∗^	Kong et al., 2007

Actinobacteria	Propionibacteriaceae	*Micropruina*	Shintani et al., 2000
		sbr-gs28^∗^	Kong et al., 2001
	Nakamurellaceae	*Nakamurella*	Yoshimi et al., 1996
	Dermatophilaceae	*Kineosphaera*	Liu et al., 2000, 2002

*^∗^Based on the MiDAS taxonomy (McIlroy et al., 2015b). ^∗∗^Not annotated in the MiDAS taxonomy as only a single representative sequence is present in the database.*

## Results and Discussion

### Overall Composition of the Microbial Communities

**FIGURE 2:**
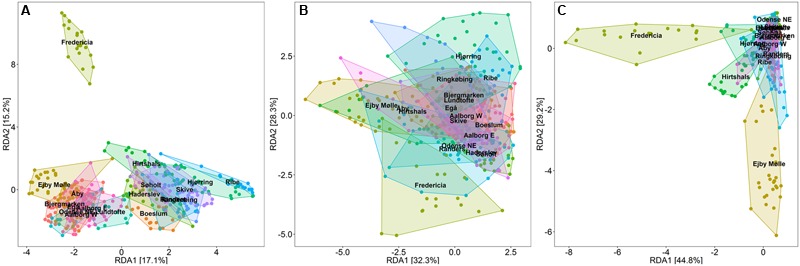
**Redundancy analysis (RDA) plots, constrained to visualize the variance in OTU abundance, explained by sample plant origin.** Axes show the percent of the total variation described by each principal component. **(A)** RDA plot of the overall bacterial community of 18 Danish full-scale EBPR WWTPs over a period of 9 years showing profiles of each plant. **(B)** RDA plot of the PAO part of the community showing similar community composition across the WWTPs, and **(C)** GAO part of the community showing dissimilarities among the WWTPs with several unique plant profiles.

The overall microbial populations in the Danish WWTPs are visualized in a redundancy analysis (RDA) plot for the entire period from 2006 to 2014 (**Figure 2A**). Despite a large shared community of abundant genera among the plants (Saunders et al., 2016), clustering of samples from each system is evident and is largely due to differences in the relative abundance of these shared genera. Interestingly, this suggests that the community composition of each plant was relatively stable throughout the 9 years—making this study, to our knowledge, the first to document such long-term stability of full-scale WWTPs.

Principal component analysis (PCA) analyses of the putative PAO and GAO populations in these plants indicate that these communities were relatively similar both within and between the plants (**Figures 2B,C**). Noted exceptions were plants receiving a high industrial load (>50%, e.g., Fredericia), primarily due to differences in the GAO populations. It is worth noting that despite relatively high levels of putative GAO in some plants (>20%), all plants have had stable P removal over several years, as previously reported (Mielczarek et al., 2013). PAO populations in general appeared to be more stably present (**Figure 2B** and Supplementary Material), where the intermittently high abundances of GAO were likely related to fluctuations in the influent COD: P ratio—where the GAO utilize excess COD (Tu and Schuler, 2013; Law et al., 2016). Though EBPR failure in full-scale systems has been related to high GAO numbers (Saunders et al., 2003), it is yet to be shown that it is directly the result of the GAO outcompeting the PAO for resources. Thus, more work is required to assess the competition hypothesis, which will importantly involve surveys that also cover inefficient and failed EBPR plants.

**FIGURE 3:**
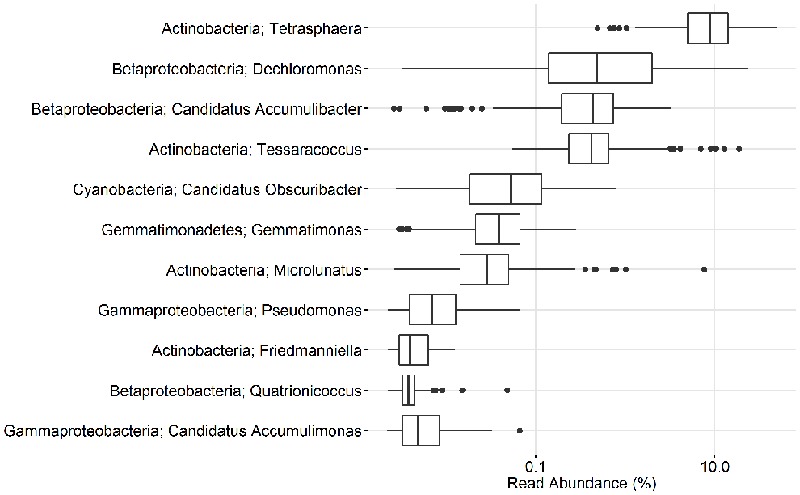
**Overview of the abundances of putative PAO.** Each box shows the data for all samples collected from 18 Danish WWTPs during the years 2006–2014. *Malikia* and *Lampropedia* were never detected. Phylum and genus are shown. Small constant (0.01) was added to all the abundance values in order to enable visualization of 0 values on the log scale.

**FIGURE 4:**
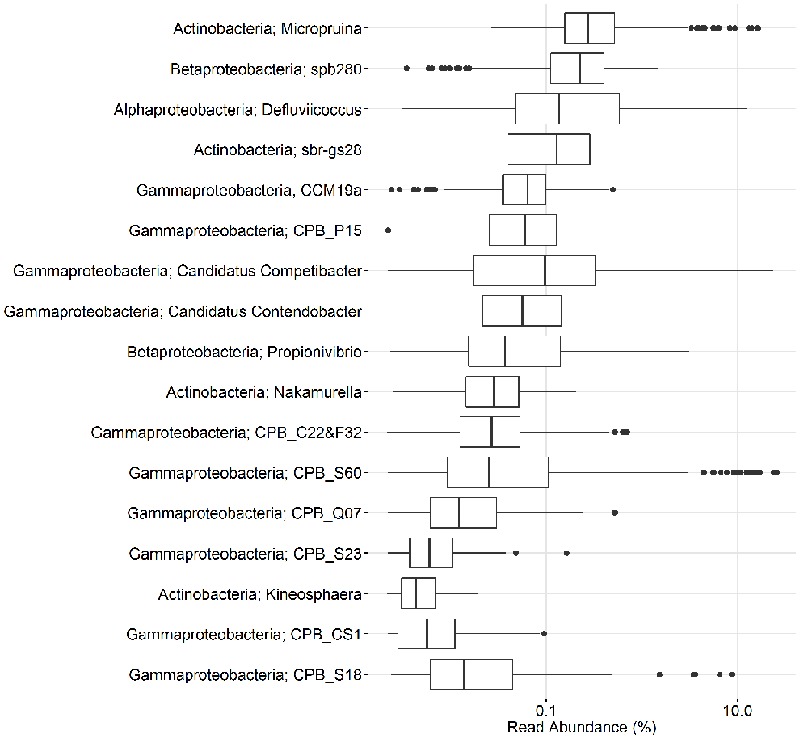
**Overview of the abundances of putative GAO.** Each box shows the data for all samples collected in 18 Danish WWTPs during the years 2006–2014. CPB_M38 is not included as it is not covered by the MiDAS taxonomy. Phylum and genus are shown. Small constant (0.01) was added to all the abundance values in order to enable visualization of 0 values on the log scale.

**FIGURE 5:**
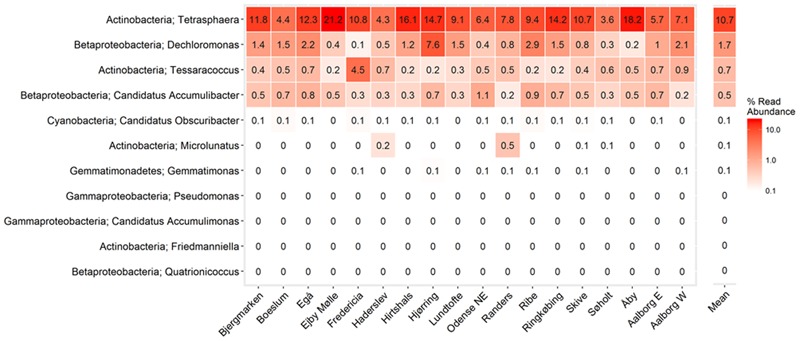
**Heatmap showing mean read abundance of putative PAOs in percent of all amplicon reads.** The numbers represent the average read abundance for given WWTP. The survey was performed in the years 2006–2014 with up to four samples per year for each plant. Phylum and genus are shown.

**FIGURE 6:**
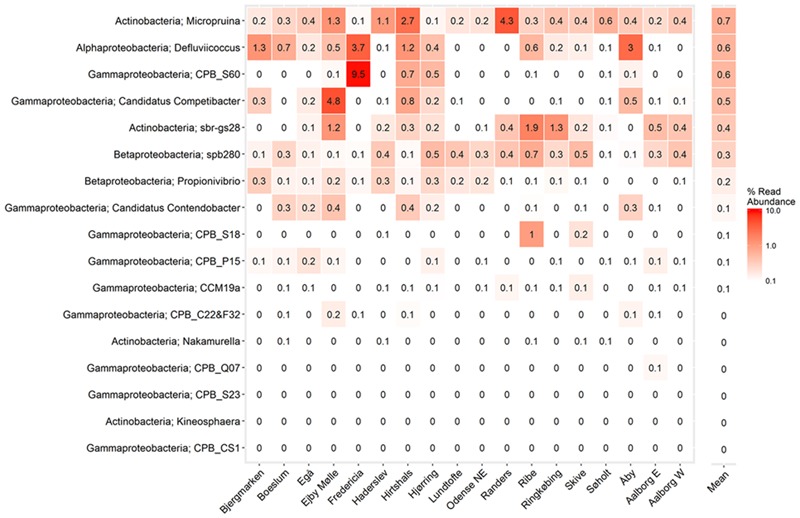
**Heatmap showing mean read abundance of putative GAOs in percent of all amplicon reads.** The numbers represent the average read abundance for the given WWTP. The survey was performed in the years 2006–2014 with up to four samples per year for each plant. Phylum and genus are shown.

In the current study, population sizes of each of the proposed PAO and GAO genera varied greatly (**Figures 3–6**). Some were consistently observed at high abundances, i.e., the *Tetrasphaera* PAO, while for others high abundance was more transient, i.e., the Competibacteraceae GAO (**Figures 3, 4**). Time series of all PAOs and GAOs in the individual plants are shown in the Supplementary Material. Several putative PAO and GAO genera were rarely detected. In light of these calculated amplicon-based abundances, evidence for a potential role in EBPR for these genera is discussed.

### The Putative PAOs

#### The Betaproteobacterial PAO

The uncultured “*Ca.* Accumulibacter” is the most studied of the PAO genera and is often assumed to be the most important PAO. The classical PAO phenotype has been demonstrated for members of the genus with both *in situ* methods (Kong et al., 2004) and in sequencing batch reactor (SBR) enrichments, where they can be enriched up to 96% of the biomass (Lu et al., 2006). For a detailed review of their physiology, see He and McMahon (2011). In this study, “*Ca.* Accumulibacter” had an average and median read abundance of 0.5 and 0.4%, respectively (**Figures 3, 5**). This is in contrast to previous FISH-based studies, including those assessing the same Danish plants, which recorded values of 3–8% (Mielczarek et al., 2013). A lack of probe specificity for the commonly applied PAOmix probe (see later) may contribute to this discrepancy (Albertsen et al., 2016). In addition, the “*Ca.* Accumulibacter” often possess relatively large cells (i.e., 2–3 μm (Kim et al., 2010), which results in relatively higher biovolume values in quantitative FISH (qFISH) analyses.

There were three MiDAS OTUs (>97% sequence similarity) classified to the “*Ca.* Accumulibacter” genus co-existing at similar abundances across the full-scale plants of this study. Several studies have investigated diversity of the genus (Martín et al., 2006; Flowers et al., 2013; Kim et al., 2013; Oyserman et al., 2015; Skennerton et al., 2015; Camejo et al., 2016). The resolution of the 16S rRNA gene to provide meaningful delineation of the genus is questionable and the polyphosphate kinase gene (*ppk*) has instead been suggested to provide this (McMahon et al., 2007). Two “*Ca.* Accumulibacter” types (I and II) are further delineated into 13 clades (IA-E and IIA-H) (He et al., 2007; Peterson et al., 2008; Mao et al., 2015). Clades reportedly vary in their morphology, carbon source, and denitrification genes (Flowers et al., 2009; Skennerton et al., 2015). Different “*Ca.* Accumulibacter” species have been suggested to vary in their level of reliance on polyphosphate storage and carbon uptake kinetics, where the predominance of a particular *ppk*-clade type may be associated with P removal efficiency (Slater et al., 2010; Welles et al., 2015, 2016). Whether or not important traits can be consistently associated with a *ppk*-type is yet to be demonstrated. Interestingly, several studies have shown that the “*Ca.* Accumulibacter” PAO can adopt the GAO phenotype under certain conditions, such as P limitation (Barat et al., 2006, 2008; Zhou et al., 2008; Welles et al., 2015, 2016), and the presence of “*Ca.* Accumulibacter” cells in full-scale EBPR plants not accumulating polyphosphate has also been reported widely (Zilles et al., 2002a,b; Kong et al., 2004; Wong et al., 2005; Beer et al., 2006). Such findings complicate the use of monitoring PAO phylotype abundances as a measure of EBPR health. Moreover, results of FISH studies should be assessed with caution, given the commonly applied FISH probe sets for the “*Ca.* Accumulibacter” (PAOmix) also target the *Propionivibrio* GAO that can be present in comparable abundances (Albertsen et al., 2016).

The second most abundant putative PAO genus in this study was *Dechloromonas*, with an average plant read abundance of 1.7%, median of 0.5%, and maximum of over 20% for some samples (**Figures 3, 5**). Contrary to the situation with the closely related “*Ca.* Accumulibacter,” *Dechloromonas* appears to be overestimated by amplicon sequencing, where values can be as much as **10**-fold higher than with FISH-based quantification (McIlroy et al., 2016; Ziegler et al., 2016). A recent isolate of the genus, from a full-scale nutrient removal system, was shown to accumulate relatively high levels of polyphosphate in axenic culture, although no further characterization was reported (Terashima et al., 2016). Members where shown *in situ* to accumulate polyphosphate and PHA, and take up carbon under anaerobic conditions (Kong et al., 2007). While some members of the genus *Dechloromonas* have been shown to behave according to the PAO phenotype, others have been shown to potentially behave as their GAO competitors (Ahn et al., 2007; McIlroy et al., 2016). What proportion of the genus are behaving as PAO, and whether or not this can be confidently assigned to any of the three abundant OTUs of this study, is not known.

#### The Putative Gammaproteobacterial PAO

Members of the gammaproteobacterial class have also long been suggested as PAO in full-scale systems (Beer et al., 2006). Members of the genus *Pseudomonas* were identified as being among the most abundant PAO in an SBR EBPR system by applying a polyphosphate staining method with fluorescence activated cell sorting (FACS; Günther et al., 2009). The low abundance of the genus in the present study (**Figure 3**) is supported by previous FISH surveys of Danish systems and they are unlikely important PAO in full-scale plants (Nguyen et al., 2012).

Also within the class Gammaproteobacteria, the uncultured “*Ca*. Accumulimonas” store carbon as PHA under anaerobic conditions and polyphosphate under subsequent aerobic conditions, which is consistent with a PAO phenotype (Nguyen et al., 2012). However, they were not abundant in the MiDAS survey (**Figures 3, 5**), despite FISH surveys reporting their consistent abundance up to 6% of the biovolume (Nguyen et al., 2012). The V1-3 primer set applied in this study was shown to give relatively poor coverage of the Gammaproteobacteria (Albertsen et al., 2015). However, a previous survey of Danish WWTPs applying the V4 primer set also failed to detect the genus in abundance (Saunders et al., 2016)—despite the primer set covering the database sequences of the “*Ca.* Accumulimonas spp.” well. Further work is required to verify their abundance, and subsequent importance in EBPR systems.

#### The Actinobacterial PAO

**FIGURE 7:**
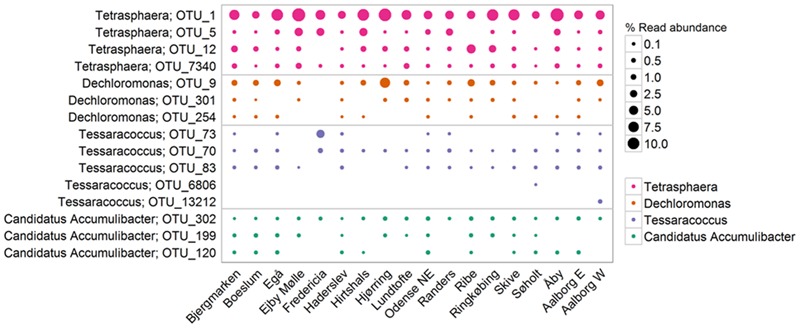
**The most abundant OTUs within each PAO-related genus.** Percentages shown represent the average read abundance of all samples collected in 18 Danish WWTPs during the years 2006–2014.

Consistent with previous qFISH-based studies, *Tetrasphaera* had the highest abundance of the PAO, with an average and median read abundance of 10.7 and 8.9%, respectively, across all plants and present in excess of 40% of the biomass in some plants. Relatively good support for the *Tetrasphaera* spp. as PAOs has been demonstrated for members of the genus in pure culture and *in situ* with polyphosphate cycling coupled to anaerobic carbon uptake (Kong et al., 2005; Nguyen et al., 2011; Kristiansen et al., 2013). However, the *Tetrasphaera* genus has demonstrated diverse physiology, including an ability for fermentative growth, and to accumulate fermentation by-products and amino acids intracellularly (Nguyen et al., 2015). Although numerically the most abundant PAO, key questions need to be answered to establish the contribution members of the genus make to P removal, i.e., how much P is accumulated relative to the “*Ca.* Accumulibacter” and how varied this is across the abundant *Tetrasphaera* spp. Three FISH probe-defined clades have been characterized *in situ* revealing differences in morphology and carbon source uptake (Nguyen et al., 2011). However, analyses of these FISH probes against the current SILVA database suggest that they are unable to separate the different clades with reasonable specificity (data not shown). Although a single dominant OTU made up most of the genus across all plants, several OTUs classified to the genus *Tetrasphaera* (**Figure 7**). This is consistent with the observations of Saunders et al. (2016). Due to limitations in the resolution of the short 16S rRNA amplicons, these OTUs could not be related to the proposed three clades of Nguyen et al. (2011).

The genus *Microlunatus* had a median read abundance of only <0.1%, but was seen as high as 7% in some plants (**Figure 3**) and has also been detected at abundances up to 9% with qFISH (Beer et al., 2006). *Microlunatus* may thus be important for the EBPR process in some plants. *Microlunatus phosphovorus* was isolated from activated sludge and shown to accumulate large amounts of polyphosphate aerobically, and release it anaerobically with a concomitant uptake of glucose (Nakamura et al., 1991, 1995b) and glutamate (Ubakata and Takii, 1994, 1998). Furthermore, *in situ* strains stain positive for polyphosphate storage (Kawaharasaki et al., 1999; Beer et al., 2006). As with the *Tetrasphaera*, in pure culture the *M. phosphovorus* can ferment sugars, and accumulate fermentation products and amino acids anaerobically (Santos et al., 1999). Although some unidentified storage polymer was postulated, neither PHA nor glycogen cycling occurred and P and carbon metabolism were not exclusively coupled (Santos et al., 1999). The type strain reportedly lacks the genes for PHA synthesis (Kawakoshi et al., 2012), however, it has been reported to synthesize the storage polymer (Akar et al., 2006). The physiology of *Microlunatus* spp. is likely quite similar to that of the *Tetrasphaera* spp. but requires further investigation.

Members of the actinobacterial genus *Tessaracoccus* were found to be relatively abundant in some plants with a median read abundance of 0.4% and up to 19% of the reads (**Figure 3**). Evidence for their role in EBPR rests solely on the ability of *T. bendigoensis* to accumulate polyphosphate in pure culture (Maszenan et al., 1999b) and nothing is known of their *in situ* physiology. Isolated members of the genus are facultative anaerobes and thus likely have a fermentative metabolism in wastewater systems (Maszenan et al., 1999b; Finster et al., 2009). Their high abundance appears to be maintained by immigration from the influent wastewater, suggesting a low growth and activity rate (Saunders et al., 2016). It is therefore unknown whether or not they play any substantial role in EBPR systems.

It has also been suggested that members of the genus “*Candidatus* Microthrix” were responsible for the bulk of P removal in a lab-scale EBPR system, which was based on positive staining for intracellular polyphosphate stores and the observed low abundance of “*Ca.* Accumulibacter” and *Tetrasphaera* spp. (Wang J. et al., 2014). Polyphosphate accumulation has been demonstrated for pure culture and *in situ* species, although it is generally thought to be a response to cell stress (Erhart et al., 1997; Tandoi et al., 1998b) and does not appear to be linked to anaerobic: aerobic carbon cycling (Andreasen and Nielsen, 2000). The genome of “*Ca.* Microthrix parvicella RN1” also lacks the low-affinity inorganic phosphate transporter (Pit) (McIlroy et al., 2013), which is theoretically essential to the PAO phenotype (Saunders et al., 2007; McIlroy et al., 2014). Its contribution to P removal is therefore uncertain; noting that regardless of any impact it may have on P removal, its presence in the system is undesirable given its proliferation is linked to the serious operational problems of bulking and foaming (Rossetti et al., 2005).

#### Other Putative PAO Isolates

Members of the genera representing putative PAO isolates, *Gemmatimonas, Friedmaniella, Quatrionicoccus, Malikia*, and *Lampropedia* were rarely detected in the Danish plants (**Figures 3, 5**) and are unlikely numerically important to the EBPR process. It should also be noted that, with the exception of *L. hyalina* (Stante et al., 1997), the only evidence suggesting their role as PAO is an ability to store visible polyphosphate granules.

A recent study hinted at the involvement of the uncultured cyanobacteria “*Candidatus* Obscuribacter phosphatis” in EBPR, based on the annotation of its genome (Soo et al., 2014). The genome possesses the low-affinity inorganic phosphate transporter (Pit), which is not in itself evidence for the PAO phenotype (McIlroy et al., 2014), given that it is also found in non-PAO organisms (e.g., *Defluviicoccus* GAO; Wang Z. et al., 2014). It is generally difficult to identify putative PAOs or GAOs based on their genome, given that the storage of polyphosphate, glycogen, and PHA are widely phylogenetically dispersed traits (Oyserman et al., 2015). *In situ* work or gene expression studies are required to assess the activities of this genus in EBPR, noting that it is also not numerically important in full-scale EBPR systems in Denmark (**Figures 3, 5**).

### Distribution of the Putative GAOs

#### The Gammaproteobacterial GAO

What was for many years considered as the genus “*Candidatus* Competibacter” has recently been suggested to be the family Competibacteraceae to better reflect the phylogenetic diversity of the group (with >89% 16S rRNA gene sequence similarity between members) (McIlroy et al., 2015a). The family is delineated by 14 FISH probe-defined clades and includes the genera “*Ca.* Competibacter,” “*Candidatus* Contendobacter,” and *Plasticicumulans*. The *Plasticicumulans* do not show the GAO phenotype and are rarely detected in full-scale plants with amplicon or qFISH analyses (McIlroy et al., 2015a), although a related phylotype was suggested to outcompete the “*Ca.* Accumulibacter” PAO in a novel EBPR system with continuous aeration (Schroeder et al., 2009). The most abundant of the groups in this study were the “*Ca.* Competibacter” and CPB_S60, which is consistent with qFISH-based studies (McIlroy et al., 2015a). Most of the genera had median abundances of less than 0.1%, yet periodically reached relatively high abundances of up to 25% in some plants (**Figures 4, 6**)—mainly those receiving high industrial loads (>50%) (i.e., Fredericia and Ejby Mølle; **Table 1**). The CPB_C22&F32, CPB_CS1, CPB_Q07, and CPB_S23 clades were never observed in read abundances greater than 1% and are unlikely important in Danish EBPR plants. The CPB_M38 clade was not assessed in this study as it is not covered by the MiDAS taxonomy given that it is represented by a single 16S rRNA gene sequence.

The Competibacteraceae are most frequently studied with family-level FISH probes and the classical GAO physiology has been confirmed with enrichment cultures (Lemos et al., 2007; Saunders et al., 2007; Oehmen et al., 2010) and *in situ* (Kong et al., 2006). Anaerobic carbon storage of VFAs as PHA in the absence of P cycling, consistent with the GAO phenotype, has also been demonstrated *in situ* for the “*Ca*. Competibacter” (formerly GB1), “*Ca*. Contendobacter” (GB5-7), CPB_S18 (GB2-3), CPB_S60 (GB4), and CPB_C22&F32 (GB8) phylotypes (Kong et al., 2002, 2006; Kim et al., 2011). All appear to have an affinity for VFAs with known differences between the phylotypes including carbon substrates type, fermentation, and denitrification (Kong et al., 2006; McIlroy et al., 2014).

Also within the Gammaproteobacteria are members of the uncultured CCM19a phylotype. These organisms were shown to store carbon as PHA under anaerobic conditions in the absence of polyphosphate storage, which is consistent with the GAO phenotype (Kong et al., 2007). In this study, they were rarely detected, with an average abundance of <0.1% and never in excess of 0.5% in any sample (**Figure 4**). Similarly, low abundances were also found with qFISH surveys, where they were up to 4% but typically <1% (Kong et al., 2007).

#### The Alphaproteobacterial GAO

**FIGURE 8:**
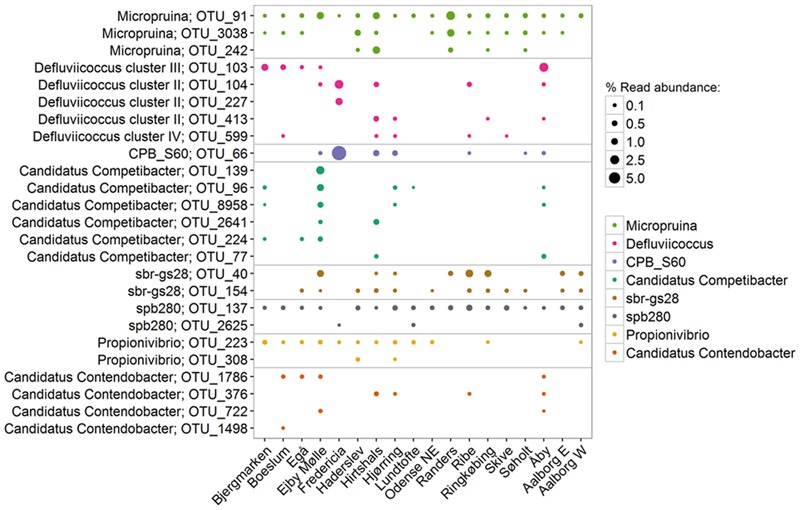
**Most abundant OTUs within each GAO-related genus.** Percentages shown represent the average read abundance of all samples collected in 18 Danish WWTPs during the years 2006–2014.

In the current study, the genus *Defluviicoccus* had a median abundance of only 0.1%, but was observed at up to 13% of the amplicon reads (**Figure 4**). Four probe-defined sub-groups currently delineate the *Defluviicoccus* genus (Wong et al., 2004; Wong and Liu, 2007; McIlroy and Seviour, 2009; Nittami et al., 2009). Interestingly, clusters 1, 2, and 4 have a TFO morphology, while cluster 3 is filamentous and has been implicated in sludge settleability problems known as bulking (Nittami et al., 2009). Clusters 1 and 2 are the most studied and have been shown to conform to the GAO model with enrichment cultures (Dai et al., 2007; Lemos et al., 2007; Burow et al., 2008, 2009; Oehmen et al., 2010) and *in situ* (Wong et al., 2004; Meyer et al., 2006; Burow et al., 2007; Wong and Liu, 2007). GAO phenotype has also been demonstrated for the sole isolate, *D. vanus*, a member of cluster 1 (Maszenan et al., 2005; Wong and Liu, 2007). Anaerobic carbon storage as PHA has also been shown *in situ* for clusters 3 and 4 (McIlroy et al., 2010). In this study, OTUs representing clusters 2 and 3 made up the majority (>90%) of amplicon reads assigned to the genus, which is consistent with FISH analyses of full-scale systems (**Figure 8**; Burow et al., 2007; McIlroy and Seviour, 2009). The original report of an alphaproteobacterial *Sphingomonas*-related GAO (Beer et al., 2004) was later shown to be incorrect and the FISH probes were shown to be binding to members of the *Defluviicoccus* cluster 1 (McIlroy et al., 2011).

#### The Betaproteobacterial GAO

The newly discovered *Propionivibrio* GAO is closely related to the “*Ca.* Accumulibacter” and is covered by the PAOmix probe set (Crocetti et al., 2000) commonly applied to target the former (Albertsen et al., 2016). In a lab-scale SBR fed with VFAs as the sole carbon source, they reached 35% of the biovolume and were shown to cycle PHA without polyphosphate storage, consistent with a GAO phenotype. In the current study, the *Propionivibrio* GAO was detected in a median read abundance of <0.1% and was present in most plants reaching abundances of up to 3% (**Figure 4**). Amplicon-based assessment of their abundance in full-scale systems has been shown to be comparable to qFISH (Albertsen et al., 2016).

The uncultured betaproteobacterial spb280, similar to the CCM19a phylotype, was also identified in full-scale systems at qFISH abundances up to 6% and the GAO phenotype partially shown *in situ* (Kong et al., 2007). The phylotype was consistently present with the second highest median abundance and maximum amplicon read abundances of 1.5% (**Figure 4**). Like the CCM19a phylotype, nothing more has been determined for the phylotype since its initial description.

#### The Actinobacterial GAO

A few isolates of the phylum Actinobacteria have been reported as potential competitors of the PAO. These include *N. multipartita* (Yoshimi et al., 1996), *M. glycogenica* (Shintani et al., 2000), and *K. limosa* (Liu et al., 2000, 2002), all shown to accumulate carbohydrates anaerobically without any polyphosphate storage. Of the genera these represent, only the *Micropruina*, along with the closely related uncultured sbr-gs28 phylotype, have been shown *in situ* to store both acetate and glucose as PHA without polyphosphate storage, consistent with the GAO phenotype (Kong et al., 2001).

In the current study, only the *Micropruina* and sbr-gs28, both members of the family Propionibacteriaceae, were found in Danish WWTPs. These had median abundances of 0.3 and 0.1%, respectively, but were present up to 16 and 5% of amplicon reads, respectively (**Figure 4**). Interestingly, the *Micropruina* were the most abundant of all putative GAOs considered in this study, based on both median and mean values (**Figures 4, 6**). Their high abundance in full-scale systems was also supported by qFISH surveys in Japan where they constituted up to 8% of the biovolume (Wong et al., 2005). Despite their relatively high abundances, little is known of their *in situ* physiology. As such, investigating their role in EBPR systems should therefore be a priority and is currently underway (McIlroy et al., unpublished).

### Implications of Metabolic Diversity among the PAO and GAO

The ability of the GAO to compete with the PAO for resources is central to their perceived importance in EBPR. As such, it is worth noting that the putative co-existing PAOs and GAOs discussed here are metabolically diverse and will not all compete for the same niche—with prominent differences related to carbon source type taken up and their ability to denitrify and/or ferment. For example, the *Defluviicocus* GAO will likely utilize the by-products of the fermentative *Tetrasphaera* PAO, rather than compete for resources. More work is required to determine the nature and importance of competition across the niches in which it occurs.

The genus is often assumed to represent a metabolically coherent group, however, several studies have shown that substantial functional diversity also exist among members of the same genus (see earlier), which can have implications for the way the microbial community composition is interpreted. The co-existence of often several closely related species of PAOs and GAOs is possibly maintained by metabolic differences supporting niche differentiation. In this study, such diversity is evident, with the abundance of each genus being made up of multiple OTUs. The level of diversity for each genus varies. For example, the “*Ca.* Accumulibacter” had three OTUs that where present in most plants at similar abundances where the genus *Tetrasphaera* had a single OTU dominating across all plants (**Figure 7**). Whether or not important traits can be associated with such 16S rRNA gene-defined OTUs in a genus is unclear and needs to be investigated further. It appears possible to identify the filamentous *Defluviicoccus* cluster 3, but not for the “*Ca.* Accumulibacter”—where the *ppk*-gene is instead suggested for phylogenetic resolution (McMahon et al., 2007). This becomes particularly important when utilizing total PAO abundances to assess EBPR health—where it suggested that some members of the genus might instead be behaving according to the GAO phenotype or fermenting without polyphosphate storage.

## Conclusion

The abundance and diversity of all proposed PAOs and GAOs were assessed in 18 Danish full-scale wastewater treatment plants with EBPR over a period of 9 years using 16S rRNA gene amplicon sequencing. The plants seem to exhibit relatively high temporal stability in the overall microbial community composition over the 9-year period. Members of the genera *Tetrasphaera*, “*Ca.* Accumulibacter”, and *Dechloromonas* are confirmed to represent the important known PAOs in full-scale EBPR systems, although abundances of the latter two appear to be heavily biased with amplicon-based methods. Potential roles for the *Microlunatus*, “*Ca.* Accumulimonas”, and *Tessaracoccus* are currently unclear and need further work. All other putative PAO considered in this study appear to be numerically unimportant in EBPR systems. For the GAOs, substantial evidence is available for the physiology of the *Defluviicoccus* and Competibacteraceae GAO only. Future work should focus on the characterization of the actinobacterial *Micropruina*, sbr-gs28, betaproteobacterial spb280, and the *Propionivibrio* phylotypes. Other phylotypes were never detected in abundance and thus are unlikely relevant in EBPR. Importantly, in this study, we only consider “known” PAOs and GAOs, which have largely been identified in lab-scale systems fed acetate or through isolation of cultures accumulating phosphate and/or carbon. Large-scale whole community surveys like MiDAS, followed by the systematic characterization of the *in situ* physiology of all abundant phylotypes in the complex full-scale EBPR systems, will likely uncover novel abundant PAOs and GAOs in the future. Phylogenetic diversity within each of the PAOs and GAOs genera varied, which is consistent with reported metabolic diversity for those tested. Whether or not key traits can be assigned to sub-genus level clades requires further investigation.

## Author Contributions

PN planned sample collection; MA, SK, and PN planned the experimental work; MA, SK, and MN performed the experimental work; MS-B, MN, MA, and SM performed the data analyses; SM, MS-B, and PN drafted the manuscript. All authors were involved in revising the manuscript.

## Conflict of Interest Statement

The authors declare that the research was conducted in the absence of any commercial or financial relationships that could be construed as a potential conflict of interest.

## Footnotes

1 http://midasfieldguide.org

2 www.midasfieldguide.org/en/protocols

3 http://www.rstudio.com/

4 https://github.com/MadsAlbertsen/ampvis

5 www.midasfieldguide.org
