# Supplementary material for: A Critical Assessment of the Microorganisms Proposed to be Important to Enhanced Biological Phosphorus Removal in Full-Scale Wastewater Treatment Systems
Source: Front Microbiol. 2017 Apr 27;8:718. doi: 10.3389/fmicb.2017.00718 (PMC5406452; doi:10.3389/fmicb.2017.00718)

Supplementary – time series

A critical assessment of the microorganisms proposed to be important to enhanced biological phosphorus removal in full-scale wastewater treatment systems

Mikkel Stokholm-Bjerregaard†, Simon J. McIlroy†, Marta Nierychlo, Søren M. Karst, Mads Albertsen, Per Halkjær Nielsen*

Center for Microbial Communities, Department of Chemistry and Bioscience, Aalborg University, Aalborg, Denmark

Time series for PAOs and GAO for 18 full-scale Danish EBPR plants 2006-2014.

Bjergmarken

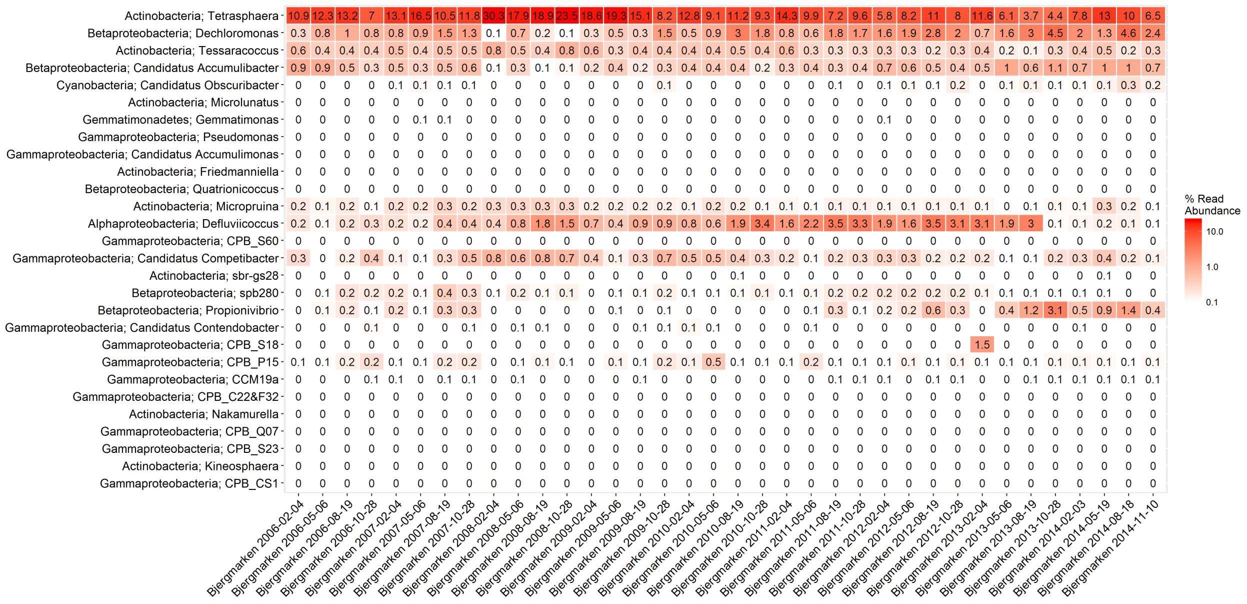

Boeslum

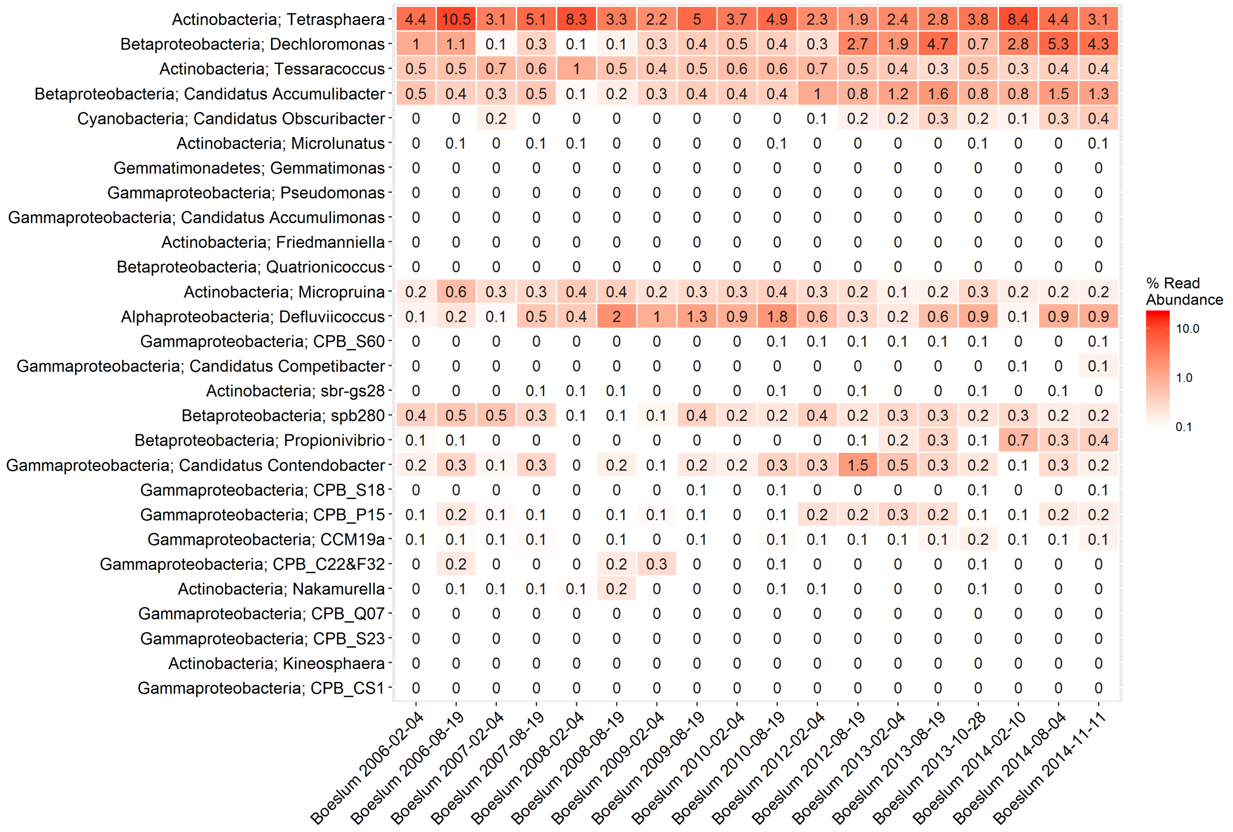
Actinobacteria; Tetrasphaera 4.4 10.5 3.1 5.1 8.3 3.3 2.2 5 3.7 4.9 2.3 1.9 2.4 2.8 3.8 8.4 4.4 3.1
Betaproteobacteria; Dechloromonas 1 1.1 0.1 0.3 0.1 0.1 0.3 0.4 0.5 0.4 0.3 2.7 1.9 4.7 0.7 2.8 5.3 4.3
Actinobacteria; Tessaracoccus 0.5 0.5 0.7 0.6 1 0.5 0.4 0.5 0.6 0.6 0.7 0.5 0.4 0.3 0.5 0.3 0.4 0.4
Betaproteobacteria; Candidatus Accumulibacter 0.5 0.4 0.3 0.5 0.1 0.2 0.3 0.4 0.4 0.4 1 0.8 1.2 1.6 0.8 0.8 1.5 1.3
Cyanobacteria; Candidatus Obscuribacter 0 0 0.2 0 0 0 0 0 0 0 0.1 0.2 0.2 0.3 0.2 0.1 0.3 0.4
Actinobacteria; Microlunatus 0 0.1 0 0.1 0.1 0 0 0 0 0.1 0 0 0 0 0.1 0 0 0.1
Gemmatimonadetes; Gemmatimonas 0 0 0 0 0 0 0 0 0 0 0 0 0 0 0 0 0 0
Gammaproteobacteria; Pseudomonas 0 0 0 0 0 0 0 0 0 0 0 0 0 0 0 0 0 0
Gammaproteobacteria; Candidatus Accumulimonas 0 0 0 0 0 0 0 0 0 0 0 0 0 0 0 0 0 0
Actinobacteria; Friedmanniella 0 0 0 0 0 0 0 0 0 0 0 0 0 0 0 0 0 0
Betaproteobacteria; Quatrionicoccus 0 0 0 0 0 0 0 0 0 0 0 0 0 0 0 0 0 0
Actinobacteria; Micropruina 0.2 0.6 0.3 0.3 0.4 0.4 0.2 0.3 0.3 0.4 0.3 0.2 0.1 0.2 0.3 0.2 0.2 0.2
Alphaproteobacteria; Defluviicoccus 0.1 0.2 0.1 0.5 0.4 2 1 1.3 0.9 1.8 0.6 0.3 0.2 0.6 0.9 0.1 0.9 0.9
Gammaproteobacteria; CPB_S60 0 0 0 0 0 0 0 0 0 0.1 0.1 0.1 0.1 0.1 0.1 0 0 0.1
Gammaproteobacteria; Candidatus Competibacter 0 0 0 0 0 0 0 0 0 0 0 0 0 0 0 0.1 0 0.1
Actinobacteria; sbr-gs28 0 0 0 0.1 0.1 0.1 0 0 0 0.1 0 0.1 0 0 0.1 0 0.1 0
Betaproteobacteria; spb280 0.4 0.5 0.5 0.3 0.1 0.1 0.1 0.4 0.2 0.2 0.4 0.2 0.3 0.3 0.2 0.3 0.2 0.2
Betaproteobacteria; Propionivibrio 0.1 0.1 0 0 0 0 0 0 0 0 0 0.1 0.2 0.3 0.1 0.7 0.3 0.4
Gammaproteobacteria; Candidatus Contendobacter 0.2 0.3 0.1 0.3 0 0.2 0.1 0.2 0.2 0.3 0.3 1.5 0.5 0.3 0.2 0.1 0.3 0.2
Gammaproteobacteria; CPB_S18 0 0 0 0 0 0 0 0.1 0 0.1 0 0 0 0 0.1 0 0 0.1
Gammaproteobacteria; CPB_P15 0.1 0.2 0.1 0.1 0 0.1 0.1 0.1 0 0.1 0.2 0.2 0.3 0.2 0.1 0.1 0.2 0.2
Gammaproteobacteria; CCM19a 0.1 0.1 0.1 0.1 0 0.1 0 0.1 0 0.1 0.1 0.1 0.1 0.1 0.2 0.1 0.1 0.1
Gammaproteobacteria; CPB_C22&F32 0 0.2 0 0 0 0.2 0.3 0 0 0.1 0 0 0 0 0.1 0 0 0
Actinobacteria; Nakamurella 0 0.1 0.1 0.1 0.1 0.2 0 0 0 0.1 0.1 0 0 0 0.1 0 0 0
Gammaproteobacteria; CPB_Q07 0 0 0 0 0 0 0 0 0 0 0 0 0 0 0 0 0 0
Gammaproteobacteria; CPB_S23 0 0 0 0 0 0 0 0 0 0 0 0 0 0 0 0 0 0
Actinobacteria; Kineosphaera 0 0 0 0 0 0 0 0 0 0 0 0 0 0 0 0 0 0
Gammaproteobacteria; CPB_CS1 0 0 0 0 0 0 0 0 0 0 0 0 0 0 0 0 0 0
Boeslum 2006-02-04
Boeslum 2006-08-19
Boeslum 2007-02-04
Boeslum 2007-08-19
Boeslum 2008-02-04
Boeslum 2008-08-19
Boeslum 2009-02-04
Boeslum 2009-08-19
Boeslum 2010-02-04
Boeslum 2010-08-19
Boeslum 2012-02-04
Boeslum 2012-08-19
Boeslum 2013-02-04
Boeslum 2013-08-19
Boeslum 2013-10-28
Boeslum 2014-02-10
Boeslum 2014-08-04
Boeslum 2014-11-11
% Read Abundance
10.0
1.0
0.1

Egå

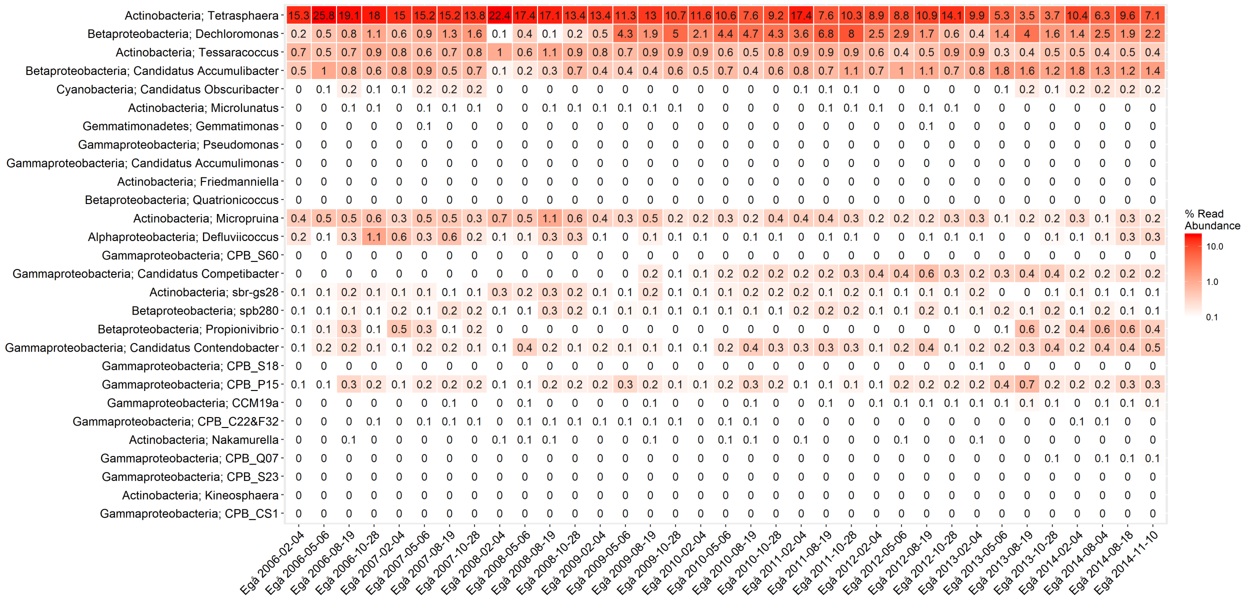
Actinobacteria; Tetrasphaera
Betaproteobacteria; Dechloromonas
Actinobacteria; Tessaracoccus
Betaproteobacteria; Candidatus Accumulibacter
Cyanobacteria; Candidatus Obscuribacter
Actinobacteria; Microlunatus
Gemmatimonadetes; Gemmatimonas
Gammaproteobacteria; Pseudomonas
Gammaproteobacteria; Candidatus Accumulimonas
Actinobacteria; Friedmanniella
Betaproteobacteria; Quatrionicoccus
Actinobacteria; Micropruina
Alphaproteobacteria; Defluviicoccus
Gammaproteobacteria; CPB_S60
Gammaproteobacteria; Candidatus Competibacter
Actinobacteria; sbr-gs28
Betaproteobacteria; spb280
Betaproteobacteria; Propionivibrio
Gammaproteobacteria; Candidatus Contendobacter
Gammaproteobacteria; CPB_S18
Gammaproteobacteria; CPB_P15
Gammaproteobacteria; CCM19a
Gammaproteobacteria; CPB_C22&F32
Actinobacteria; Nakamurella
Gammaproteobacteria; CPB_Q07
Gammaproteobacteria; CPB_S23
Actinobacteria; Kineosphaera
Gammaproteobacteria; CPB_CS1
% Read Abundance
10.0
1.0
0.1

Ejby mølle

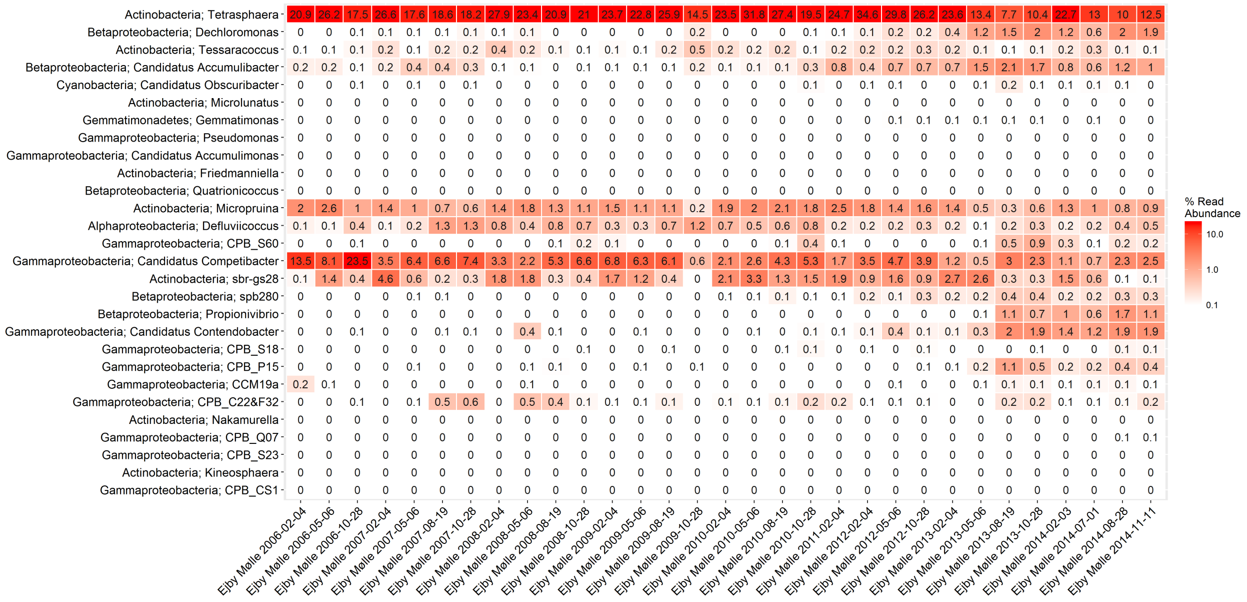

Fredericia

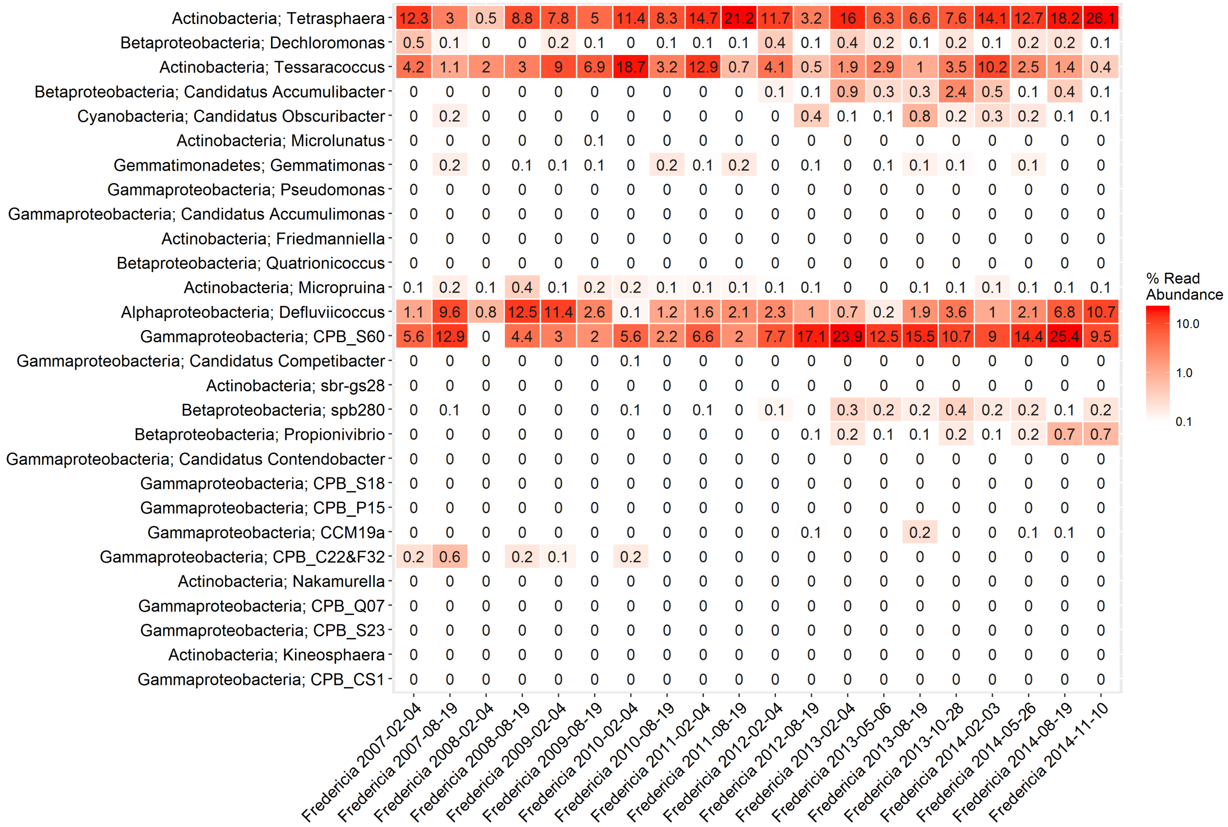

Haderslev

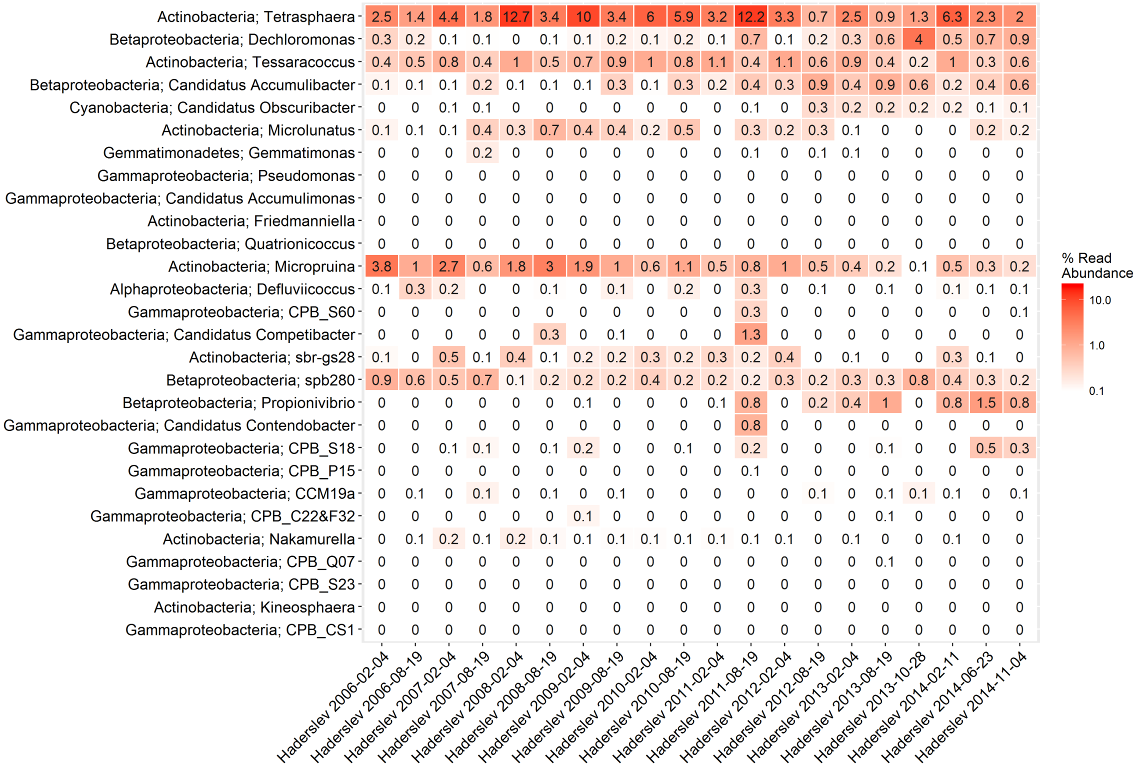

Hirtshals

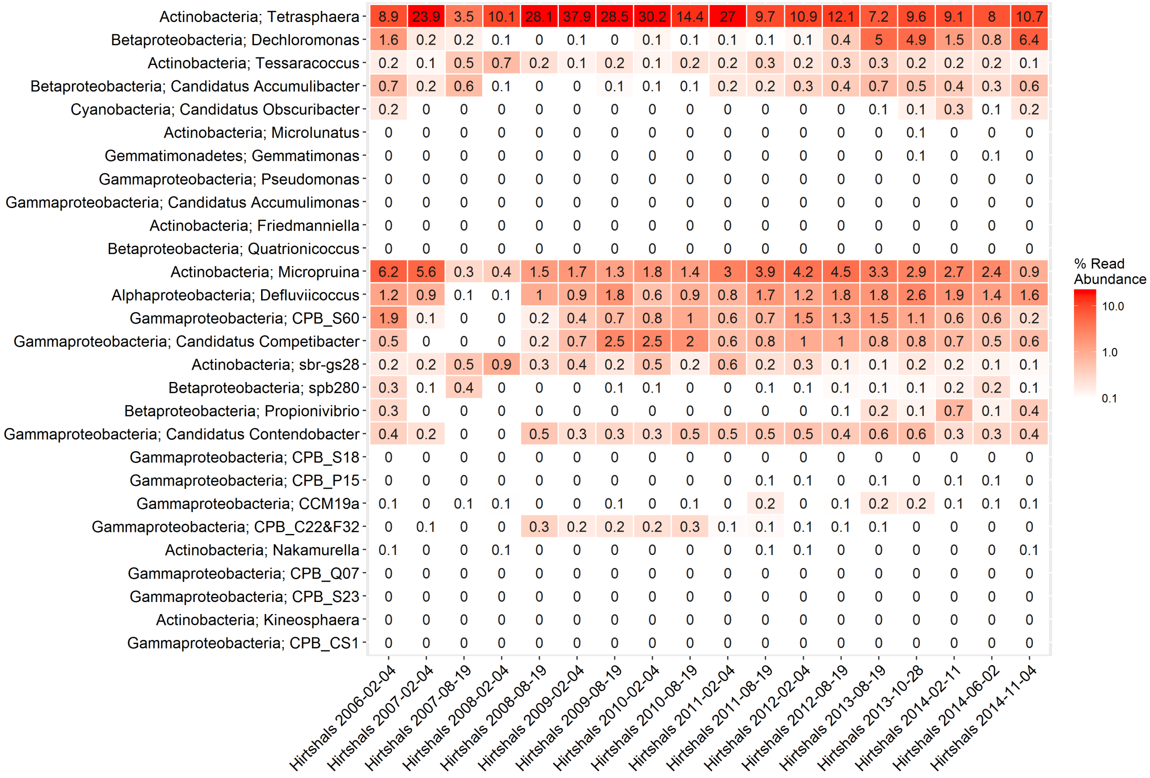

Lundtofte

	Lundtofte 2006-02-04	Lundtofte 2006-08-19	Lundtofte 2007-02-04	Lundtofte 2007-08-19	Lundtofte 2009-02-04	Lundtofte 2009-08-19	Lundtofte 2010-02-04	Lundtofte 2010-08-19	Lundtofte 2013-02-04	Lundtofte 2013-08-19	Lundtofte 2013-10-28	Lundtofte 2014-02-10	Lundtofte 2014-06-23	Lundtofte 2014-11-04
Actinobacteria; Tetrasphaera	12.4	6.7	8.9	5.9	13.6	8.4	12.7	9.4	9.7	6.8	6.4	9	10	6.8
Betaproteobacteria; Dechloromonas	0.7	0.2	0	0.1	0	0.1	0.1	0.2	2.6	2.3	5.6	3.1	1.9	3.4
Actinobacteria; Tessaracoccus	0.5	0.2	0.7	0.4	0.2	0.2	0.2	0.3	0.1	0.2	0.1	0.3	0.2	0.1
Betaproteobacteria; Candidatus Accumulibacter	0.2	0.1	0.1	0.1	0	0.1	0.2	0.2	0.5	0.5	0.6	0.9	0.4	0.5
Cyanobacteria; Candidatus Obscuribacter	0	0	0	0	0	0	0	0	0.2	0.1	0.1	0.1	0.1	0
Actinobacteria; Microlunatus	0	0	0	0	0	0	0	0	0	0	0	0	0	0
Gemmatimonadetes; Gemmatimonas	0	0	0	0	0	0	0	0	0.1	0.1	0	0	0.1	0.1
Gammaproteobacteria; Pseudomonas	0	0	0	0	0	0	0	0	0	0	0	0	0	0
Gammaproteobacteria; Candidatus Accumulimonas	0	0	0	0	0	0	0	0	0	0	0	0	0	0
Actinobacteria; Friedmanniella	0	0	0	0	0	0	0	0	0	0	0	0	0	0
Betaproteobacteria; Quatrionicoccus	0	0	0	0	0	0	0	0	0	0	0	0	0	0
Actinobacteria; Micropruina	0.4	0.2	0.4	0.3	0.3	0.2	0.3	0.4	0	0	0	0.1	0	0
Alphaproteobacteria; Defluviicoccus	0	0	0	0.1	0	0	0	0	0	0	0	0.2	0	0
Gammaproteobacteria; CPB_S60	0	0	0	0	0	0	0	0	0	0	0	0	0	0
Gammaproteobacteria; Candidatus Competibacter	0	0	0	0	0	0	0.1	0.2	0.3	0.2	0	0	0	0.1
Actinobacteria; sbr-gs28	0	0	0	0	0	0	0	0	0	0	0	0	0	0
Betaproteobacteria; spb280	0.3	0.3	0.3	0.4	0.4	0.2	0.3	0.2	0.7	0.6	0.6	0.4	0.5	0.3
Betaproteobacteria; Propionivibrio	0	0	0	0	0	0	0	0	0.1	0.2	0.8	0.5	0.5	0.3
Gammaproteobacteria; Candidatus Contendobacter	0	0	0	0	0	0	0	0	0	0	0	0.1	0	0
Gammaproteobacteria; CPB_S18	0	0	0	0	0	0	0	0	0	0	0	0	0	0
Gammaproteobacteria; CPB_P15	0	0	0	0	0	0	0	0	0	0	0	0.1	0	0
Gammaproteobacteria; CCM19a	0	0	0	0	0	0	0	0	0	0.1	0.1	0.1	0.1	0.1
Gammaproteobacteria; CPB_C22&F32	0	0	0	0	0	0	0	0	0	0	0	0	0	0
Actinobacteria; Nakamurella	0	0	0	0	0	0	0	0	0	0	0	0	0	0
Gammaproteobacteria; CPB_Q07	0	0	0	0	0	0	0	0	0	0	0	0	0	0
Gammaproteobacteria; CPB_S23	0	0	0	0	0	0	0	0	0	0	0	0	0	0
Actinobacteria; Kineosphaera	0	0	0	0	0	0	0	0	0	0	0	0	0	0
Gammaproteobacteria; CPB_CS1	0	0	0	0	0	0	0	0	0	0	0	0	0	0

% Read Abundance: 10.0, 1.0, 0.1

Odense NE

	Odense NE 2006-02-04	Odense NE 2006-08-19	Odense NE 2007-02-04	Odense NE 2007-08-19	Odense NE 2008-02-04	Odense NE 2008-08-19	Odense NE 2009-02-04	Odense NE 2009-08-19	Odense NE 2010-02-04	Odense NE 2010-08-19	Odense NE 2011-02-04	Odense NE 2011-08-19	Odense NE 2012-02-04	Odense NE 2012-08-19	Odense NE 2013-02-04	Odense NE 2013-08-19	Odense NE 2013-10-28	Odense NE 2014-02-03	Odense NE 2014-08-06	Odense NE 2014-11-11
Actinobacteria; Tetrasphaera	12.3	11.6	7	4.4	9.2	8.1	9.9	10.7	6.4	5.9	7.9	5.6	4.2	4.4	2.6	2.2	2.5	3.4	5.8	4.2
Betaproteobacteria; Dechloromonas	0.1	0.1	0.1	0.1	0	0.1	0.1	0.1	0.3	0.2	0.1	0.1	0.3	0.4	0.6	1.1	1.4	0.8	1.2	1.3
Actinobacteria; Tessaracoccus	0.2	0.3	0.7	0.3	0.9	0.6	0.3	0.6	0.4	0.7	0.5	0.6	0.4	0.3	0.2	0.2	0.4	0.4	0.4	0.2
Betaproteobacteria; Candidatus Accumulibacter	0.4	0.3	0.6	0.4	0.1	0.1	0.7	0.6	0.8	0.5	0.6	0.6	1.3	1.6	1.8	2	3.2	1.4	2.5	1.8
Cyanobacteria; Candidatus Obscuribacter	0.1	0	0	0	0	0	0	0	0	0	0	0	0	0.3	0.1	0.1	0.1	0.1	0.1	0.1
Actinobacteria; Microlunatus	0	0	0	0	0	0.1	0	0.1	0	0.1	0	0.1	0	0.1	0	0	0.1	0	0.1	0
Gemmatimonadetes; Gemmatimonas	0.1	0.1	0	0.2	0	0	0	0.1	0.1	0	0	0.1	0	0.1	0.1	0.1	0	0	0.1	0.1
Gammaproteobacteria; Pseudomonas	0	0	0.1	0	0	0	0	0	0	0	0	0	0	0	0	0	0	0	0	0
Gammaproteobacteria; Candidatus Accumulimonas	0	0	0	0	0	0	0	0	0	0	0	0	0	0	0	0	0	0	0	0
Actinobacteria; Friedmanniella	0	0	0	0	0	0	0	0	0	0	0	0	0	0	0	0	0	0	0	0
Betaproteobacteria; Quatrionicoccus	0	0	0	0	0	0	0	0	0	0	0	0	0	0	0	0	0	0	0	0
Actinobacteria; Micropruina	0.1	0.7	0.2	0.1	0.3	0.2	0.1	0.3	0.1	0.2	0.2	0.3	0.2	0.1	0.1	0.2	0.2	0.2	0.2	0.1
Alphaproteobacteria; Defluviicoccus	0	0	0	0	0	0.1	0	0	0	0.1	0	0	0	0	0	0	0	0	0.1	0.1
Gammaproteobacteria; CPB_S60	0	0	0	0	0	0	0	0	0	0	0	0	0	0	0	0	0	0	0	0
Gammaproteobacteria; Candidatus Competibacter	0	0	0	0	0	0	0	0	0	0	0	0	0	0	0	0	0	0	0	0
Actinobacteria; sbr-gs28	0	0	0.1	0	0.1	0.1	0	0.1	0.1	0.1	0.1	0.1	0.1	0	0	0	0.1	0.1	0.1	0
Betaproteobacteria; spb280	0.2	0.4	0.2	0.3	0	0.1	0.2	0.3	0.2	0.2	0.3	0.4	0.2	0.4	0.4	0.6	0.2	0.3	0.2	0.1
Betaproteobacteria; Propionivibrio	0	0.1	0	0	0	0	0	0	0	0	0.1	0	0	0.1	0.1	0.5	0.4	0.5	1	0.6
Gammaproteobacteria; Candidatus Contendobacter	0	0.1	0	0	0	0	0	0	0	0	0	0	0	0	0	0	0	0	0	0
Gammaproteobacteria; CPB_S18	0	0	0	0	0	0	0	0	0	0	0	0	0	0	0	0	0	0	0	0
Gammaproteobacteria; CPB_P15	0	0	0	0	0	0.1	0.1	0.2	0	0	0.1	0	0	0.2	0.1	0.1	0.1	0	0.2	0.2
Gammaproteobacteria; CCM19a	0.1	0.1	0	0.1	0	0	0	0	0	0	0	0.1	0	0.1	0.1	0.1	0	0	0	0
Gammaproteobacteria; CPB_C22&F32	0	0.2	0	0	0	0.1	0	0	0.1	0.1	0	0	0	0	0	0	0	0	0.1	0.1
Actinobacteria; Nakamurella	0	0	0	0	0.1	0	0	0	0	0.1	0	0.1	0.1	0	0	0	0.1	0	0	0
Gammaproteobacteria; CPB_Q07	0	0	0	0	0	0.1	0	0	0	0	0	0	0	0	0	0	0	0	0	0.1
Gammaproteobacteria; CPB_S23	0	0	0	0	0	0	0	0	0	0	0	0	0	0	0	0	0	0	0	0
Actinobacteria; Kineosphaera	0	0	0	0	0	0	0	0	0	0	0	0	0	0	0	0	0	0	0	0
Gammaproteobacteria; CPB_CS1	0	0	0	0	0	0	0	0	0	0	0	0	0	0	0	0	0	0	0	0

% Read Abundance: 10.0, 1.0, 0.1

Randers

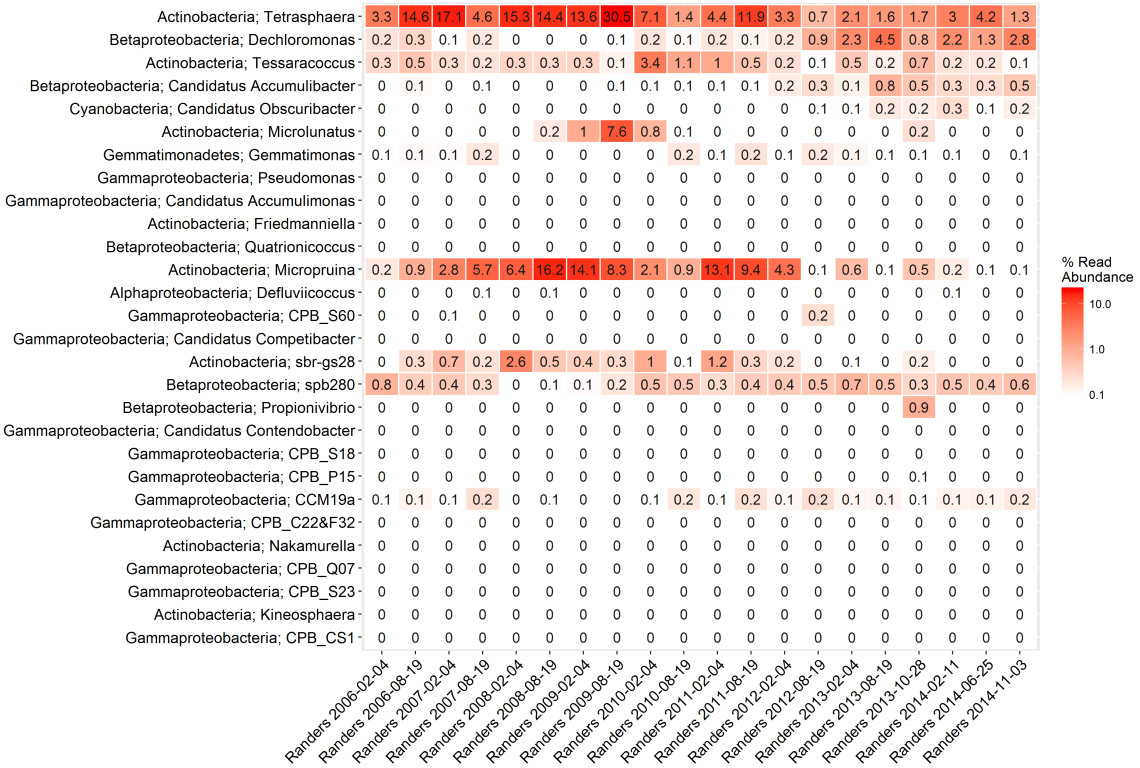

Ribe

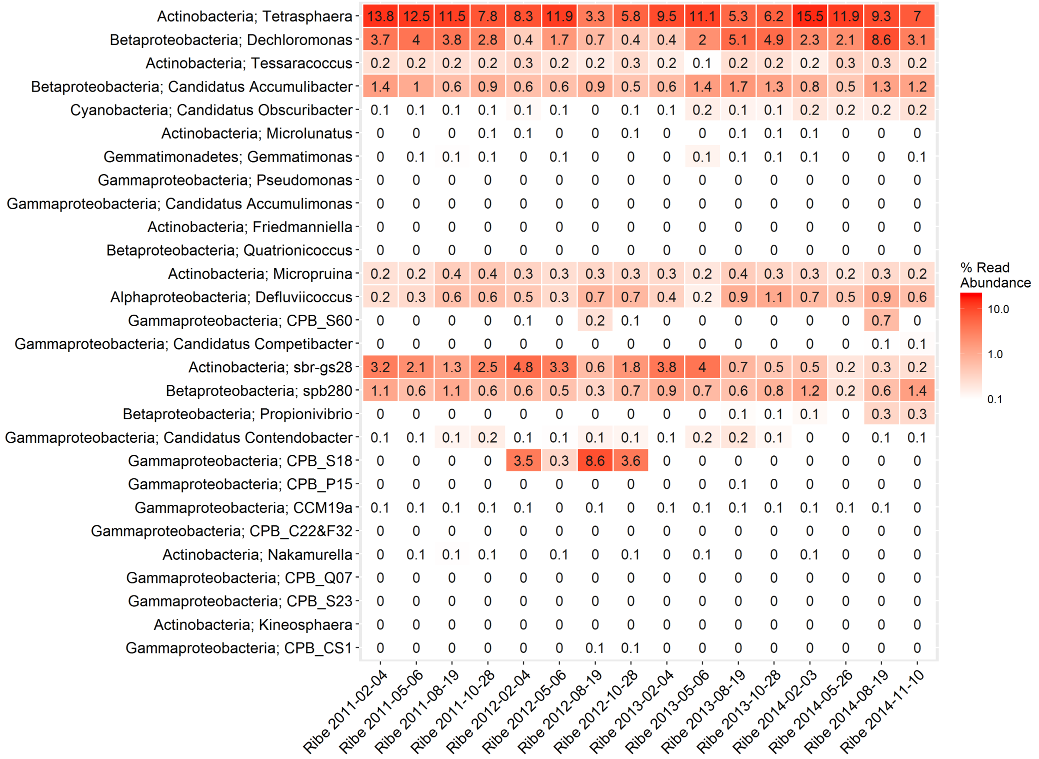

Ringkøbing

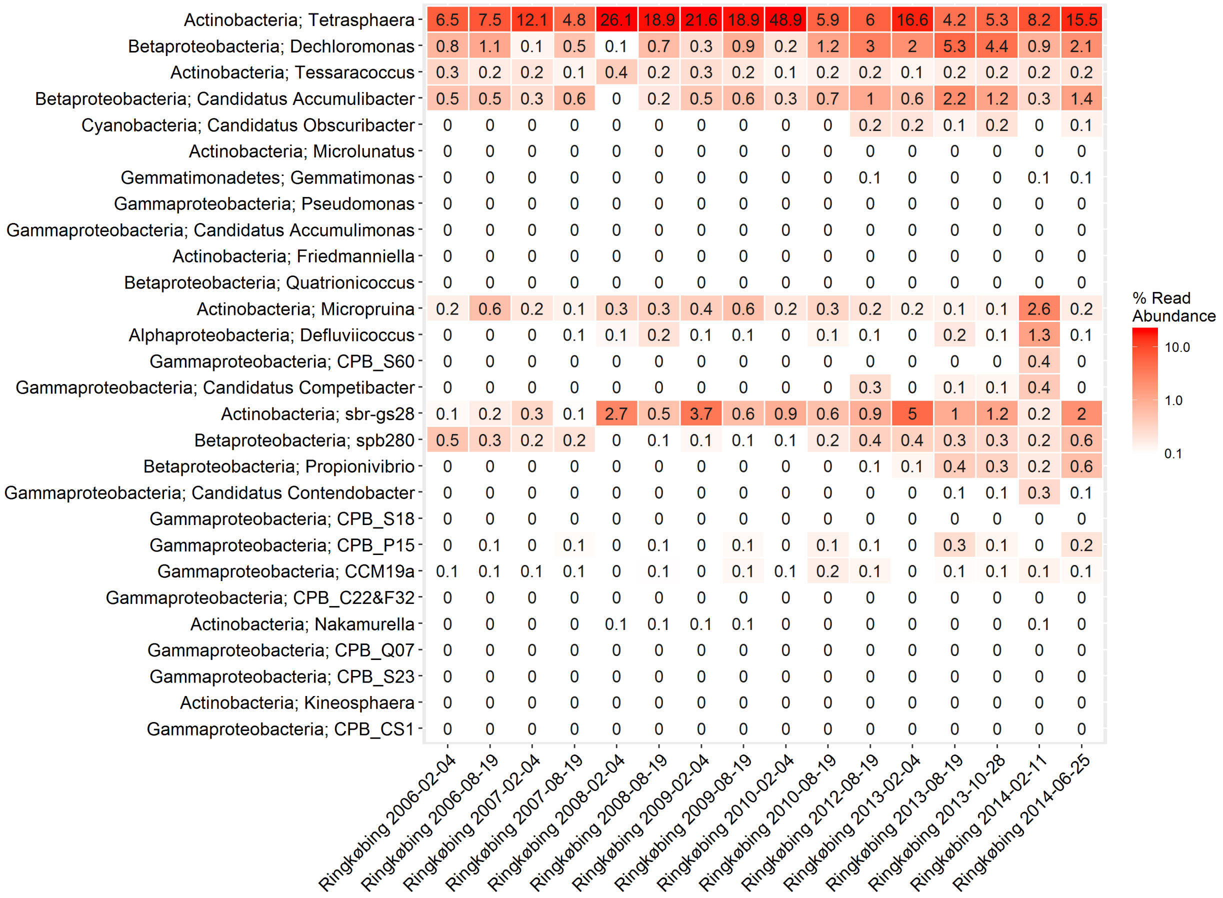

Skive

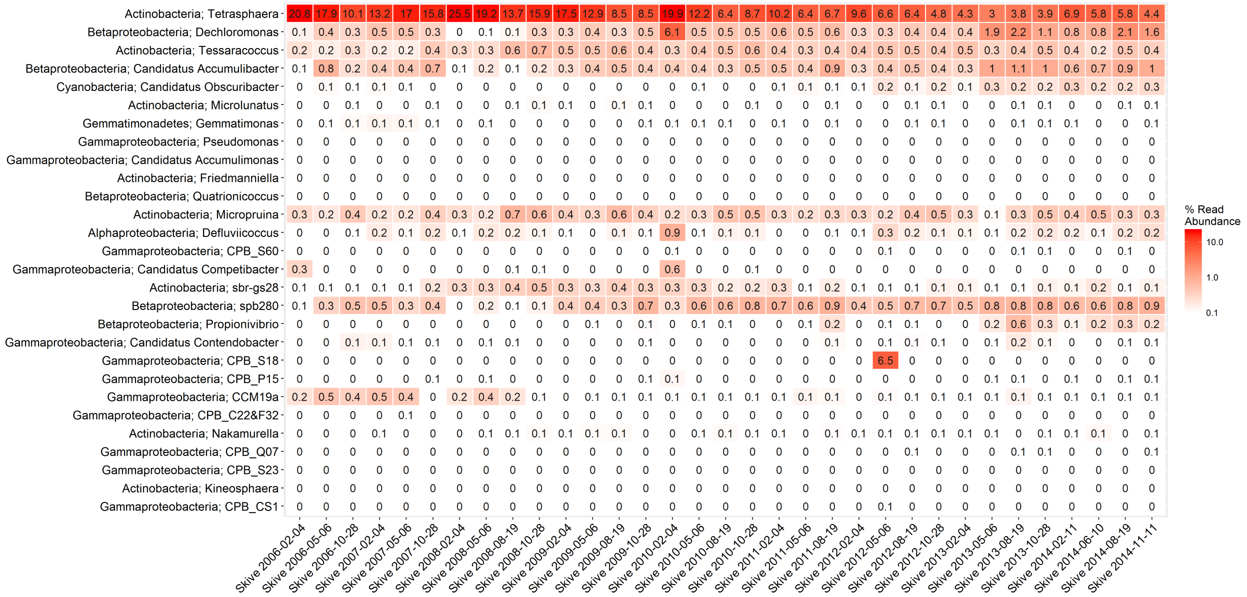

Søholt

	Søholt 2006-02-04	Søholt 2006-08-19	Søholt 2007-02-04	Søholt 2007-08-19	Søholt 2008-02-04	Søholt 2009-02-04	Søholt 2009-08-19	Søholt 2010-02-04	Søholt 2010-08-19	Søholt 2011-02-04	Søholt 2011-08-19	Søholt 2012-08-19	Søholt 2013-02-04
Actinobacteria; Tetrasphaera	4.9	1	2.5	0.8	4.3	4.1	3.3	3.2	5.6	4.7	6.9	1.6	3.5
Betaproteobacteria; Dechloromonas	0.2	0.1	0.1	0.1	0.1	0.2	1.1	0.3	0.6	0.6	0.1	0.2	0.8
Actinobacteria; Tessaracoccus	0.5	0.4	0.8	0.6	1	0.6	0.5	0.5	0.6	0.4	0.6	0.6	0.2
Betaproteobacteria; Candidatus Accumulibacter	0.7	0.3	0.3	0.2	0.1	0.1	0.4	0.3	0.3	0.6	0.3	0.1	0.4
Cyanobacteria; Candidatus Obscuribacter	0.2	0.1	0.2	0.1	0	0	0	0.1	0	0.1	0	0	0.1
Actinobacteria; Microlunatus	0.1	0.1	0.1	0.1	0	0.1	0.1	0	0.1	0	0.1	0.1	0
Gemmatimonadetes; Gemmatimonas	0	0	0	0	0	0	0	0	0	0	0	0	0
Gammaproteobacteria; Pseudomonas	0	0	0	0	0	0	0	0	0	0	0	0	0
Gammaproteobacteria; Candidatus Accumulimonas	0	0	0	0	0	0	0	0	0	0	0	0	0
Actinobacteria; Friedmanniella	0	0	0	0	0	0	0	0	0	0	0	0	0
Betaproteobacteria; Quatrionicoccus	0	0	0	0	0	0	0	0	0	0	0	0	0
Actinobacteria; Micropruina	0.4	0.4	0.5	0.5	0.8	0.7	0.6	0.9	0.9	1.1	0.8	0.4	0.3
Alphaproteobacteria; Defluviicoccus	0	0.1	0	0.1	0.1	0	0.1	0	0.1	0	0.1	0.3	0
Gammaproteobacteria; CPB_S60	0	0.1	0	0.1	0	0.1	0.1	0	0	0	0	0.3	0
Gammaproteobacteria; Candidatus Competibacter	0	0	0	0	0	0	0.1	0	0	0	0	0.4	0
Actinobacteria; sbr-gs28	0.1	0	0.1	0.1	0.2	0.2	0.1	0.2	0.2	0.1	0.1	0.1	0.1
Betaproteobacteria; spb280	0	0	0	0	0	0	0.1	0.1	0.1	0.1	0.1	0	0.5
Betaproteobacteria; Propionivibrio	0	0	0	0	0	0	0	0	0	0.1	0	0	0.2
Gammaproteobacteria; Candidatus Contendobacter	0	0	0	0	0	0	0	0	0	0	0	0.1	0
Gammaproteobacteria; CPB_S18	0	0	0	0	0	0	0	0	0	0	0	0	0
Gammaproteobacteria; CPB_P15	0	0	0	0	0	0.2	0.1	0.1	0.2	0.1	0.2	0.1	0
Gammaproteobacteria; CCM19a	0	0	0	0.1	0	0	0	0	0	0	0.1	0	0.1
Gammaproteobacteria; CPB_C22&F32	0	0	0	0.1	0	0	0.1	0	0	0	0	0.1	0
Actinobacteria; Nakamurella	0.1	0.1	0.1	0.1	0.1	0.1	0.1	0	0.1	0.1	0.1	0.1	0.1
Gammaproteobacteria; CPB_Q07	0	0	0	0.1	0	0	0	0	0	0	0	0.1	0
Gammaproteobacteria; CPB_S23	0	0	0	0	0	0	0.2	0	0	0	0	0	0
Actinobacteria; Kineosphaera	0	0	0	0	0	0	0	0	0	0	0	0	0
Gammaproteobacteria; CPB_CS1	0	0	0	0	0	0	0	0	0	0	0	0	0

% Read Abundance: 10.0, 1.0, 0.1

Aby

	Åby 2006-02-04	Åby 2006-08-19	Åby 2007-02-04	Åby 2007-08-19	Åby 2008-02-04	Åby 2008-08-19	Åby 2009-02-04	Åby 2009-08-19	Åby 2010-02-04	Åby 2010-08-19	Åby 2013-02-04
Actinobacteria; Tetrasphaera	30.4	9.5	24	6.4	39.5	19.9	25.4	10.5	12.7	16.1	5.6
Betaproteobacteria; Dechloromonas	0.3	0.1	0.1	0.1	0	0	0.1	0.1	0.1	0.2	1.1
Actinobacteria; Tessaracoccus	0.4	0.4	0.5	0.6	0.9	0.5	0.8	0.5	0.5	0.5	0.3
Betaproteobacteria; Candidatus Accumulibacter	0.8	0.7	0.5	0.4	0	0.1	0.3	0.3	0.7	0.3	1
Cyanobacteria; Candidatus Obscuribacter	0	0	0	0	0	0	0	0	0	0	0.1
Actinobacteria; Microlunatus	0	0.1	0	0.1	0	0.1	0.1	0	0	0	0
Gemmatimonadetes; Gemmatimonas	0	0	0	0	0	0	0	0	0	0	0
Gammaproteobacteria; Pseudomonas	0	0	0	0	0	0	0	0	0	0	0
Gammaproteobacteria; Candidatus Accumulimonas	0	0	0	0	0	0	0	0	0	0	0
Actinobacteria; Friedmanniella	0	0	0	0	0	0	0	0	0	0	0
Betaproteobacteria; Quatrionicoccus	0	0	0	0	0	0	0	0	0	0	0
Actinobacteria; Micropruina	0.3	0.3	1	0.5	0.5	0.3	0.3	0.2	0.2	0.3	0.1
Alphaproteobacteria; Defluviicoccus	1.5	3.2	1.2	7.5	2.9	3	1.5	3.8	2	5	1.4
Gammaproteobacteria; CPB_S60	0.2	0.7	0.1	0.4	0	0	0	0	0	0	0
Gammaproteobacteria; Candidatus Competibacter	0.8	1.4	0.3	0.7	0.2	0.7	0.4	0.5	0.3	0.4	0
Actinobacteria; sbr-gs28	0	0	0	0.1	0.1	0	0	0	0	0	0
Betaproteobacteria; spb280	0.1	0.1	0.1	0.3	0	0	0.1	0.1	0.1	0.2	0.2
Betaproteobacteria; Propionivibrio	0	0	0	0	0	0	0	0	0	0	0.1
Gammaproteobacteria; Candidatus Contendobacter	0.3	1.2	0.4	0.9	0	0.1	0	0.1	0	0.1	0.1
Gammaproteobacteria; CPB_S18	0	0.1	0	0.1	0	0	0	0	0	0	0
Gammaproteobacteria; CPB_P15	0.1	0.1	0	0.1	0	0.1	0	0.1	0.1	0.1	0.1
Gammaproteobacteria; CCM19a	0	0	0	0.1	0	0	0	0	0	0	0
Gammaproteobacteria; CPB_C22&F32	0	0.1	0	0.7	0.2	0.1	0	0.1	0.1	0.1	0.1
Actinobacteria; Nakamurella	0	0	0	0	0	0	0	0	0	0	0
Gammaproteobacteria; CPB_Q07	0	0	0	0.1	0	0	0	0	0	0.1	0
Gammaproteobacteria; CPB_S23	0	0	0	0	0	0	0	0	0	0	0
Actinobacteria; Kineosphaera	0	0	0	0	0	0	0	0	0	0	0
Gammaproteobacteria; CPB_CS1	0	0	0	0	0	0	0	0	0	0	0

% Read Abundance: 10.0, 1.0, 0.1

Aalborg E

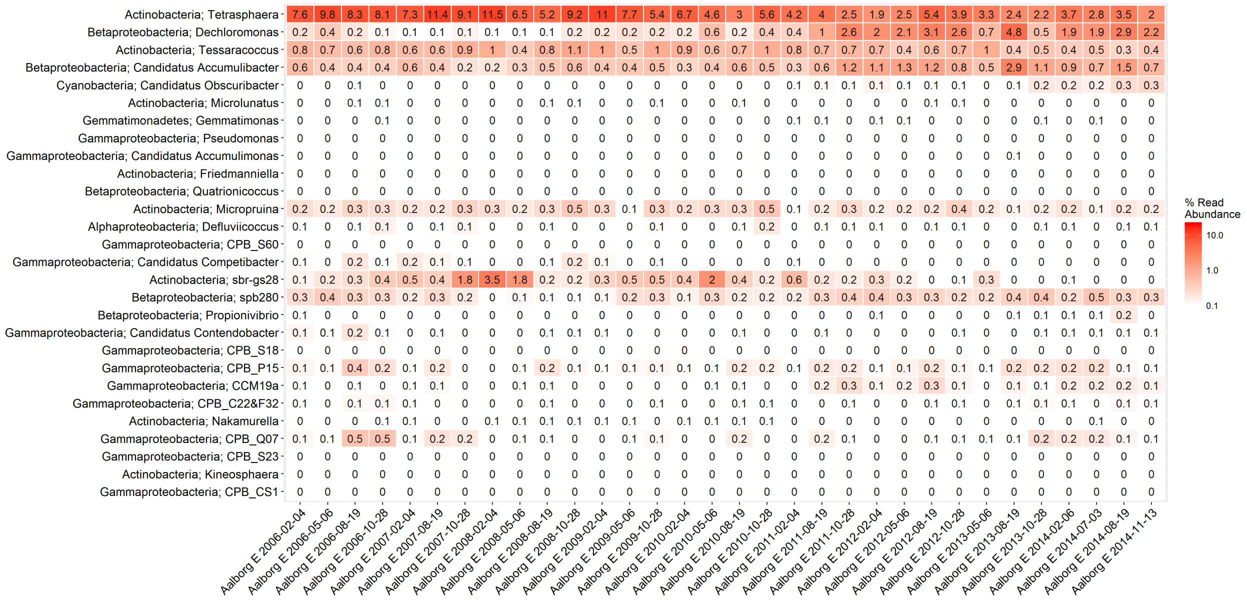

Aalborg W

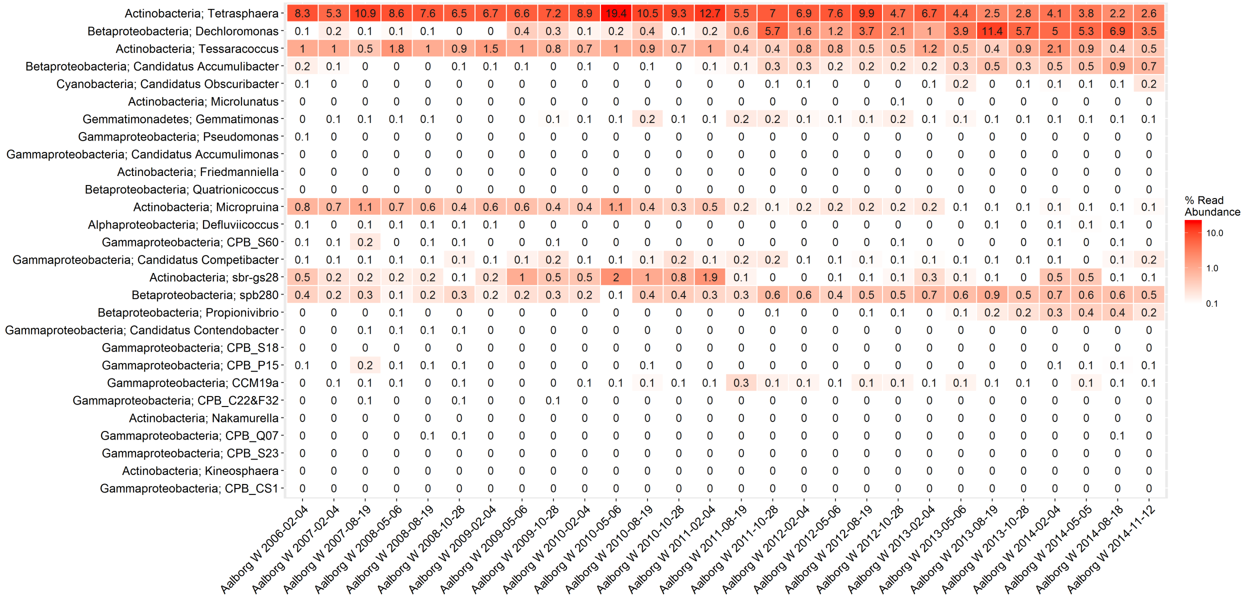

Supplement: Supplementary file 1 [file Data_Sheet_1.PDF]
